# Smart Nanotechnologies for Multimodal Neuromodulation and Brain Interfacing

**DOI:** 10.1002/advs.202524300

**Published:** 2026-03-29

**Authors:** Tommaso Curiale, Marie Celine Lefevre, Alessio Carmignani, Maria Cristina Ceccarelli, Matteo Battaglini, Attilio Marino, Gianni Ciofani

**Affiliations:** ^1^ Smart Bio‐Interfaces Istituto Italiano di Tecnologia Pontedera Italy

**Keywords:** nanosensors, neuromodulation, organic nanomaterials, smart nanotechnologies, stimuli‐responsive nanoparticles

## Abstract

This review surveys recent advances in active nanomaterials for neuromodulation, with a focus on remotely controlled nanotransducers for precise manipulation of brain functions. We discuss how stimuli‐responsive nanomaterials enable spatiotemporally precise brain interfacing through remotely controlled actuation; furthermore, we examine energy transduction mechanisms underlying nanoparticle‐assisted neuromodulation, and highlight nanosensors that monitor bioelectrical and neurochemical activity with high spatial and temporal resolution. Beyond neurons, we consider strategies targeting glial function, as well as emerging approaches to cross or bypass the blood–brain barrier. Finally, we outline key challenges for clinical translation, including long‐term safety, biointegration, and regulatory considerations. Together, these developments position smart nanotechnologies as a foundation for next‐generation precision brain interfacing, with the potential to design patient‐tailored therapeutic solutions across neurological and psychiatric disorders.

## Graphical abstract

Recent advances in smart nanotechnologies are expanding the toolbox for brain interfacing, from wireless neuromodulation and high‐resolution sensing to targeted delivery within the central nervous system. By combining responsive nanomaterials with bioinspired design, these platforms enable multimodal interactions with neurons and glia, while also interfacing with genetically targeted approaches, with growing potential for minimally invasive and clinically relevant applications.

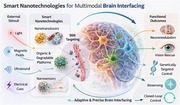

## 1 Introduction

The remarkable complexity of the brain has long challenged efforts to interface with it safely, stably, and with cellular precision. Conventional electrodes provide valuable temporal control but at the cost of invasiveness and limited cell‐type selectivity, whereas genetic tools, while intrinsically selective, face blood‐brain barrier (BBB)‐limited delivery, vector payload, immunogenicity constraints, and unresolved safety/regulatory hurdles [1]. Smart nanoparticles can be engineered to sense, integrate/transduce stimuli, trigger neural activation, and activate specific pathways in both neuronal and non‐neuronal cells. By coupling targeted transport with stimuli‐responsive behaviors, these nanoscale systems navigate immune surveillance, recognize molecular cues in the neurovascular and parenchymal niches, and transduce external energy (optical, magnetic, acoustic, or electric) into localized biochemical or bioelectric effects. Precision, in this context, means not only “where” (spatial and cell‐type specificity) but also “when” (on‐demand, feedback‐gated timing) and “what” (payload identity and mode of action), ideally while minimizing perturbation to surrounding neuronal‐glial networks [1, 2].

As a working analogy, consider a precision vehicle engineered to navigate and operate within brain tissue. The “*engine*” and “*transmission*” convert external “fuel” into the exact form of energy required locally: thermal, electrical, or mechanical cues directed at the plasma membrane or specific organelles. The “*steering*” aligns interventions to the correct biological target. The “*dashboard*” senses chemistry and state, closing the loop. The “*suspension*” damps friction and wear ‐in our case, the set of strategies that buffer immune reactivity and glial scarring over chronic use‐. The “chassis and tires” must conform to the terrain (soft, hydrated, ion‐rich tissue), and, in our case, correspond to organic nanomaterials whose chemistry and mechanics are tuned to safely grip this environment.

Organic nanoplatforms, such as conjugated polymers, conductive hydrogels, lipids, and supramolecular assemblies, can be designed to operate safely in the neural environment. Organic nanomaterials tuned to kPa‐scale elastic modulus better match the softness of neural tissue, and mitigate mechanical‐mismatch‐driven foreign‐body responses [1, 3]. Many of them are biodegradable and designed for transient function, with ester, anhydride, acetal, or enzyme‐cleavable linkers that allow the duration of material stability and activity to be programmed to match a defined therapeutic window (e.g., the period of post‐injury plasticity), after which these minimally invasive nanoimplants self‐resorb in situ according to the encoded degradation profile, without external intervention [4]. The same chemistries enable loading of theranostic agents and on‐demand release via internal cues (pH, enzymes, reactive oxygen species ‐ROS‐) or external triggers (e.g., near‐infrared ‐NIR‐ light and focused ultrasound ‐FUS‐). Beyond hosting payloads, organic nanomaterials can be designed to serve as nanotransducers, such as semiconducting polymer nanoparticles for photothermal/photoelectrochemical effects and piezo/flexoelectric phases for acoustic‐to‐field conversion. These materials constitute the organic interface that integrates sensing, stimulation, and payload delivery beyond conventional electrode functions [1, 3, 4].

In the vehicle analogy, active nanotransducers are the transmission that converts remotely delivered energy into a local stimulatory effect [5]. Light‐responsive semiconductors and plasmonic structures provide photothermal or photoelectrochemical neuromodulation; magnetic nanoparticles enable magnetothermal heating, magnetomechanical torque, or magnetoelectric field generation; ultrasound‐responsive nanoplatforms and piezoelectrics transduce acoustic pressure into localized forces or potentials. Across these modalities, the unifying principle is wireless neuromodulation with spatial selectivity spanning millimeters to single cells and subcellular structures, possibly complementing genetic strategies like opto‐ and chemogenetics where precise neural population targeting is required [6, 7]. Alongside optical, magnetic, and acoustic drives, wireless links (including magnetoelectric coupling) and in situ harvesters, like triboelectric and piezoelectric nanogenerators, shrink batteries and drive battery‐free activities [8, 9, 10, 11, 12].

Following the vehicle analogy, even the best engine fails without steering. Targeting and delivering drugs and stimuli to the brain and the right neural circuits while avoiding off‐target tissue is fundamental for precise neuromodulation. Strategies to cross or bypass the BBB, like receptor‐mediated transcytosis (RMT) (e.g., TfR/CD98hc shuttles), BBB‐crossing lipid nanoparticles (LNPs), FUS‐mediated opening with microbubbles, and magnetic guidance, now offer complementary approaches to efficiently control nanoparticles and payload delivery. In addition, the use of stimuli‐responsive release (pH‐based, enzymatic, magnetic or acoustic triggers) adds a final, local lock to the delivery key [13, 14, 15, 16, 17, 18].

Closing the loop requires nanosensors that report brain activity and status, not just electrically, but chemically. NIR I/II probes based on single‐walled carbon nanotubes (SWCNTs) visualize catecholamine dynamics with sub‐second resolution in scattering tissue, offering a non‐genetic complement to protein indicators [19]. In parallel, graphene and organic transistor sensors, together with advanced electrochemical interfaces, enable multiplex quantification of neurotransmitters and modulators on flexible, low‐impedance platforms [7]. Embedding such sensors with actuators supports adaptive dosing of energy and drugs, giving the operational definition of precision neuromodulation [7, 19].

**A**strocytes and microglia govern synaptic homeostasis, neuroimmune tone, and neurovascular coupling; glial cells are becoming increasingly important in precision brain interfacing: astrocytes and microglia modulate neural activation, neuroinflammation, and gliosis, controlling and regulating brain function. As therapeutically significant examples, it has been demonstrated that nanoparticle‐assisted regulation of brain microglia activity may restore antitumor function, while astrocyte‐focused modulation within brainstem autonomic nuclei enables precise control of cardiovascular responses during stimulation [20, 21].

Precision increases when modalities cooperate. Pairings of ultrasound‐responsive mechanoluminescent nanotransducers with opsins enable fiber‐free sono‐optogenetics while NIR‐to‐visible upconversion supports multiplexed, multicolor control with reduced thermal load in rodents. Also, nanoparticle‐assisted magnetothermal and magnetomechanical stimulation remotely activate neurons without implanted emitters. Persistent constraints remain (e.g., viral expression and intracranial placement, heating and dose limits, human‐scale field parameters), yet the synergy between nanomedicine and genetics advances brain interfacing, enabling precise control of anatomical targeting, defined activation of neural populations/subpopulations, temporal stimulation patterning, and pathway‐specific modulation [6, 22].

This review first highlights recent advances in active nanomaterials for neuromodulation, then turns to organic nanoplatforms engineered around biocompatibility and programmed degradability. We next examine the external optical, magnetic, and acoustic sources that drive their activation, and then focus on vision‐rescue strategies, in which nanotransducers restore photoreceptor function. Finally, we report on recent nanosensors for precise monitoring of neurochemical and bioelectric dynamics, alongside state‐of‐the‐art approaches for crossing the brain's protective barriers to achieve efficient delivery and targeting. In this review, we map material classes (organic, inorganic, and bio‐hybrid), discuss mechanisms that engage both neurons and non‐neuronal partners (astrocytes, microglia, and vascular cells), and evaluate translational constraints, biodegradability, long‐term safety, manufacturability, and regulatory readiness.

## 2 Advances in Active Nanomaterials for Neuromodulation

**FIGURE 1:**
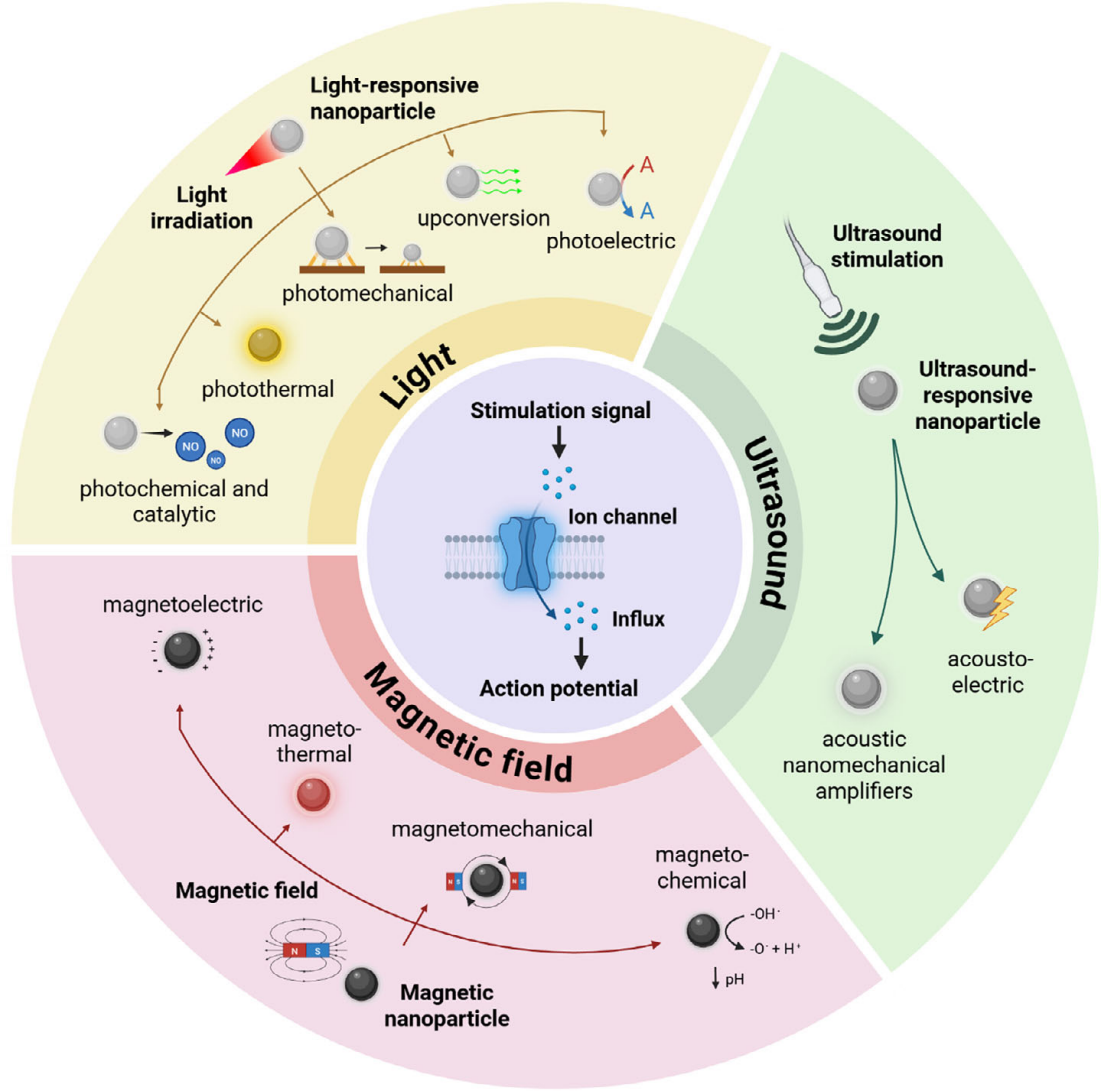
Schematic representation of nanoscale transduction mechanisms for remote neuromodulation. Light‐responsive nanoparticles convert optical irradiation into photothermal, photomechanical, photochemical/catalytic, upconversion, or photoelectric signals at the cell interface. Magnetic nanoparticles transform externally applied magnetic fields into magnetothermal, magnetomechanical, magnetochemical, or magnetoelectric outputs. Ultrasound‐responsive nanoparticles act as acoustic nanomechanical amplifiers or acousto‐electric transducers. In all cases, the generated physical or chemical cues modulate ion channels, drive ionic influx, and elicit action potentials in excitable cells. Created with BioRender.com.

Active nanomaterials represent a class of wireless neuromodulation tools that offer high precision with minimal invasiveness. These are typically nanoscale materials engineered to convert external physical stimuli (e.g., light, magnetic fields, and ultrasound) into localized effects such as heat, electrical signals, mechanical forces, or into the controlled release of chemical and biochemical agents that modulate neuronal activity [23]. The main nanoscale transduction pathways for light‐, magnetic‐, and ultrasound‐responsive nanoparticles are schematized in Figure 1. By delivering these active nanomaterials to targeted brain regions or specific cell types, researchers can remotely influence neural circuits without the need to implant electrodes or perform genetic modification [24]. This approach addresses the limitations of traditional neuromodulation (e.g., deep brain stimulation electrodes or optogenetics requiring fiber optics) by enabling minimally invasive, spatiotemporally precise, and cell‐specific neural control [25]. Below, we review key advances in various categories of active nanomaterials for neuromodulation, grouped by their underlying physical transduction mechanisms, and highlight representative examples and recent developments.

### 2.1 Neuromodulation via Light‐Activated Nanotransducers

Light is historically the most mature neuromodulatory energy source: photons strongly interact with tissue, can be focused with micrometer precision, and their intensity can be modulated on the microsecond timescale to match neuronal dynamics [26]. Accordingly, optical stimulation offers high spatial resolution and rapid on/off control of neurons; however, conventional optical neuromodulation often requires invasive fiber optics or genetic introduction of light‐sensitive proteins for deep targets [27, 28]. The main physical limitation is represented by the optical transport: visible light is scattered and absorbed so strongly that intensity can drop by orders of magnitude over a few millimetres of brain tissue [29]. By contrast, in the NIR window (∼650–1300 nm), scattering and absorption are reduced: for instance, 1070 nm light experiences ∼20‐fold attenuation at 4 mm in the mouse brain, whereas blue light is essentially extinguished over the same path [30]. Active nanomaterials aim to exploit light, particularly NIR light, to trigger physical or chemical effects at targeted sites, achieving neuromodulation without implants or transgenes. These nanomaterials can be engineered to convert light into heat, mechanical forces, electrical charges, or chemical signals [26].

**FIGURE 2:**
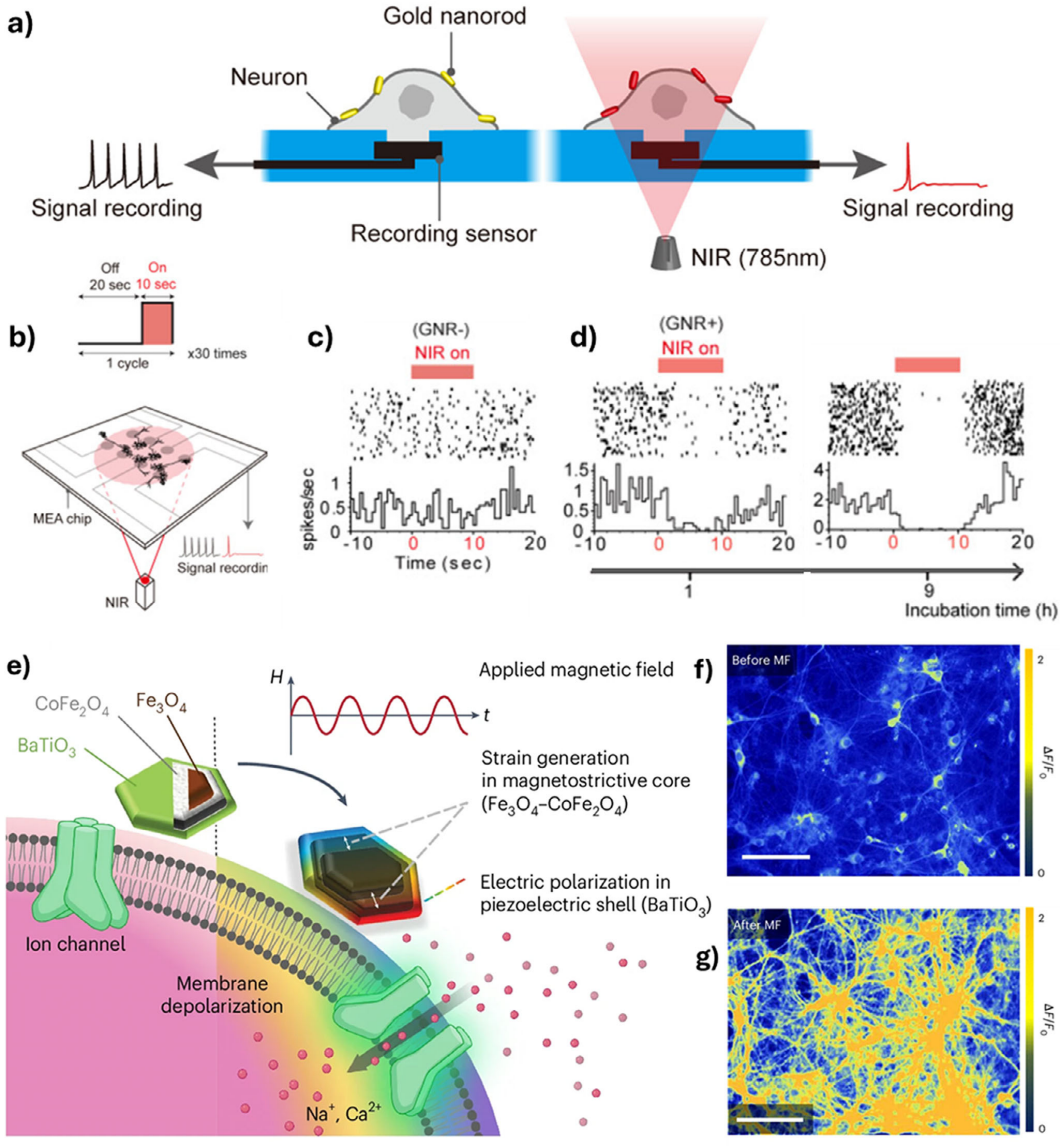
Photothermal and magnetoelectric nanotransducers for remote neuromodulation. (a–d) Photothermal modulation of neural activity. Schematic of NH_2_‐PEG‐functionalized gold nanorods (GNR) binding to neuronal membranes and converting NIR light into heat (a), and suppressing activity in neuronal cells, measured by extracellular electrodes (b). Untreated neurons upon repeated NIR irradiation (15 mW/mm^2^) show minimal response to NIR irradiation (c), while GNR‐treated neurons exhibit incubation‐time‐dependent suppression of spiking during NIR exposure (d). Adapted with permission from [32] 2014, ACS. (e–g) Magnetoelectric nanodisc (MEND)‐mediated neuronal depolarization: (e) Schematic of neuromodulation mediated by MENDs, and the relative fluorescence change (∆F/F_0_) of GCaMP6s, a genetically encoded reporter of cytosolic Ca^2+^, in hippocampal neurons decorated with MENDs before (f) and after (g) magnetic field application (10 s, oscillating magnetic field 220 mT; AMF 1 kHz, 10 mT). Scale bars, 150 µm. Adapted from with permission from [57] 2025, Nature Group.

Considering the photothermal effect, nanomaterials such as gold nanorods (AuNRs), nanoshells, carbon‐based particles, or copper sulfide nanocrystals efficiently absorb light and convert it to heat, with the possibility to stimulate temperature‐sensitive ion channels in neurons [31]. For instance, Yoo et al. attached AuNRs to neuronal membranes and showed that NIR laser stimulation (808 nm) caused a local photothermal rise that reversibly inhibited neuronal firing [32]. The heating opened in neurons K^+^ channels like TREK‐1, dampening their excitability (Figure 2a–d). Conversely, by targeting heat‐sensitive excitatory channels such as TRPV1, photothermal nanostructures can also stimulate neuronal firing [33]. Thus, by choosing appropriate ion channel targets and nanoparticle functionalization, photothermal approaches can either suppress or activate neural activity with light.

A key challenge for optical neuromodulation is that visible light, for instance, employed to directly activate opsins or other light‐sensitive targets, is strongly scattered and absorbed in tissue [34]. Upconversion nanocrystals address this issue by converting deep‐penetrating NIR light into visible light at the target site [35]. These particles absorb multiple NIR photons and then emit higher‐energy visible photons, acting as nano‐transducers that generate light within deep tissues [36]. For example, Ma et al. injected photoreceptor‐targeted upconversion nanoparticles (UCNPs) into the mouse retina and enabled the mice to detect NIR illumination as visual signals [37]. UCNPs have also been used to activate optogenetic proteins like channel rhodopsin in deep brain regions, allowing wireless neuronal stimulation with external NIR light instead of implanted fibers [30]. This strategy permits minimally invasive optical neuromodulation in deep tissue, and ongoing development of UCNPs with tunable emissions (even into ultraviolet) opens possibilities for activating diverse light‐gated pathways and inducing therapeutic biochemical events in situ.

Beyond directly modulating ion channels, light‐responsive nanomaterials can drive chemical reactions that influence neural activity. In this approach, nanoparticles act as light‐triggered catalysts or depots to locally release neuromodulators [38]. For example, a hybrid system was developed with a UCNPs‐based core and a porous polymer shell loaded with a nitric oxide donor compound. Upon NIR illumination, the UCNP emits ultraviolet radiation photons that cleave the nitric oxide donor, releasing nitric oxide on demand [39]. In a spinal cord injury model, this light‐triggered nitric oxide release promoted neuronal outgrowth and functional repair, illustrating that optical stimulation can act via a biochemical cue. Such strategies essentially turn light into a localized drug release or signaling event, expanding neuromodulation beyond purely electrical pathways.

Another approach is represented by the conversion of light into electric currents at the target site. Semiconductor or photovoltaic nanomaterials (e.g., silicon nanostructures, titanium oxide, conducting polymers) can act as tiny photo‐capacitors, generating charge separation when illuminated [40]. If these nanodevices are adjacent to neurons, the resulting electric field can depolarize the cell membrane and trigger action potentials [41]. Jiang et al. exploited TiO_2_ nanoparticles decorated with gold, showing that 405 nm light caused the nano‐composite to depolarize nearby neuron‐like cells and trigger Ca^2+^ influx [42]. This wireless stimulation mimics the effect of an electrode and can operate on microsecond timescales, and holds promise for therapeutic neuromodulation without implanted devices.

Light can also drive mechanical forces at the nanoscale. Photomechanical neuromodulation uses specially designed nanoparticles that translate optical energy into a physical force on cells, thereby activating mechanosensitive pathways [43]. Many neurons express ion channels that respond to membrane stretch or pressure (e.g., TRPA1 and TREK‐1) [44]. If a nanoparticle locally pushes or pulls the cell membrane, it can open such channels and modulate excitability [45]. Liu et al. developed an optomechanical nanotransducer consisting of a AuNR core, heating under NIR light, coated with a temperature‐responsive polymer shell. An NIR pulse causes the gold core's photothermal heating to collapse the polymer shell, exerting a mechanical tug on the membrane [46]. In cultured cells, these shrinking nanoparticles activated stretch‐sensitive receptors on the cell surface, triggering downstream signals and calcium influx. Although still in early stages, this technique demonstrates a way to engage neurons by optically pulling on ion channel complexes or membranes with subcellular precision.

Overall, light‐responsive nanomaterials constitute a rich toolkit for neuromodulation, encompassing multiple outcomes (e.g., thermal, chemical, electrical, or mechanical transduction) under optical control. Researchers have achieved outcomes ranging from reversible suppression of neural activity to restoration of vision and deep brain stimulation using light‐driven nanoparticles. Optical triggering offers very high temporal precision, since nanoparticles can be activated with microsecond light pulses, and the ability to target tiny regions with focused beams or patterned illumination; however, a major limitation is the shallow penetration of light into brain tissue [47]. This challenge has spurred the development of nanomaterial‐based strategies that harness non‐optical energy sources, such as magnetic fields and ultrasound, which can penetrate deeper into the brain.

### 2.2 Neuromodulation via Magnetically Activated Nanotransducers

Conversely to light, which is strongly absorbed and scattered by tissue, static and low‐frequency magnetic fields weakly interact with biological matter, crossing the skull and entire brain with minimal attenuation and without causing ionization [48]. As a result, magnetic fields offer a powerful tool for remote neuromodulation: they penetrate living tissue readily and noninvasively, and when applied at moderate amplitudes within appropriate frequency ranges, do not cause significant tissue heating or other direct tissue damage [49]. On their own, however, such fields barely perturb neurons because neurons lack intrinsic magnetoreceptors, therefore implementing magnetic neuromodulation requires nanomaterials that transduce magnetic fields into biologically relevant stimuli. Recent advances in magnetic nanoparticles have enabled several paradigms of magnetic neuromodulation, including heat generation (magnetothermal effect), mechanical force transfer (magnetomechanical coupling), and local electric polarization (magnetoelectric transduction), as well as indirect biochemical effects [48]. In practice, magnetic nanoparticles are delivered to a target region, and an external magnetic field is applied over the whole brain: only the nanoparticles in the target area convert the field into a neural stimulus, providing spatial specificity defined by the nanoparticle distribution.

Superparamagnetic nanoparticles, such as iron oxide or ferrite nanocrystals, can absorb energy from an alternating magnetic field (AMF) and release it as heat to stimulate heat‐sensitive ion channels in neurons [50]. In a key demonstration, Huang et al. delivered magnetic nanoparticles to mice expressing the heat‐gated channel TRPV1 in certain neurons. The application of a radio‐frequency magnetic field caused the particles to heat and open the TRPV1 channels, depolarizing those neurons [49]. This approach, sometimes termed “magnetogenetics”, achieved wireless activation of specific neurons using a magnetic trigger. Ongoing efforts aim to eliminate any genetic requirements by targeting endogenous thermosensitive channels (e.g., TREK‐1 or TRPV4) or by using magnetothermal heating to trigger local drug release near neurons [51].

Magnetic fields can exert forces or torques on magnetic particles, which then mechanically stimulate cells. For example, field gradients can pull magnetic particles against cell membranes, or rotating magnetic fields can spin anisotropic particles to twist cell membranes [52]. Early studies used micrometric‐scale magnetic beads bound to cell‐surface receptors to mechanically perturb cells in vitro, whereas more recent approaches rely on nanoscale magnetic particles to achieve finer, localized control. Lee et al. designed nanoscale magnetic discs (“m‐Torquer”) that deliver piconewton‐scale torques to neurons in vivo [45]. After these nanodiscs were injected into the mouse brain, an applied rotating magnetic field caused them to twist and mechanically open Piezo1 channels on neurons, leading to reversible changes in the animals’ behavior corresponding to neuron activation. Magnetomechanical stimulation directly engages native mechanosensitive channels, so it can potentially work without any genetic modification if target neurons naturally express those channels [53]. Additionally, mechanical stimuli may activate unique signaling pathways in cells beyond just triggering action potentials [54].

A particularly innovative approach is represented by the application of magnetoelectric nanoparticles, which convert magnetic inputs directly into local electric fields, thanks to a magnetostrictive core (e.g., cobalt ferrite) and a piezoelectric shell (e.g., barium titanate) [55]. Under an external magnetic field, the core strains and in turn strain the shell, generating an electric potential across the particle [56]. Recently, Kim et al. reported 250 nm magnetoelectric nanodiscs that could stimulate neurons with relatively weak magnetic fields [57]. When injected in the mouse brain and an oscillating magnetic field was applied, they induced neuronal firing without any genetic sensitization, working as remote‐controlled nanoelectrodes (Figure 2e,f). In the future, magnetoelectric stimulation might become a non‐invasive alternative to implanted electrodes for disorders like Parkinson's disease or epilepsy; however, translating this strategy to humans will require efficient coupling at field amplitudes and frequencies compatible with safety limits, rigorous demonstration of long‐term magnetoelectric nanoparticle biocompatibility and clearance, and robust control over off‐target activation.

Magnetic fields can also induce biochemical changes in the neural microenvironment via functional nanoparticles. For example, Park et al. coated magnetic iron oxide nanoparticles with a heat‐sensitive polymer that releases acidic molecules when warmed [58]. The application of an AMF caused the nanoparticles to produce a localized drop in pH due to the polymer's breakdown, which opened pH‐sensitive ion channels on nearby neurons, thus activating their excitation. Notably, this effect was highly localized and did not measurably heat surrounding tissue. Such examples show that magnetic inputs can remotely initiate chemical signals (like pH changes or neurotransmitter release) to modulate neuronal activity, expanding the scope of magnetic neuromodulation beyond direct thermal or electrical stimulation [59].

In summary, magnetic nanomaterials have become a major modality for precision neuromodulation, thanks to their ability to safely penetrate to deep brain regions without attenuation [60]. Magnetic stimulation is also favorable for clinical translation because static and low‐frequency magnetic fields are generally safe, as evidenced by magnetic resonance imaging technology [61]. By using nanoparticles to convert these benign fields into localized thermal, mechanical, or electrical stimuli at neural targets, researchers have created a powerful platform for remote brain stimulation. The field has quickly progressed from early approaches that required genetically sensitized neurons to newer methods, such as magnetomechanical and magnetoelectric stimulation, that directly activate neurons through native mechanisms without the need for genetic modification [62]. Magnetic nanoparticle approaches thus offer a non‐invasive neural interface with deep penetration, diverse modes of action, and promising results in modulating neural circuits and disease models.

### 2.3 Neuromodulation via Ultrasound‐Activated Nanotransducers

Ultrasound consists of pressure waves, at frequencies above the audible range. For biomedical applications, low‐frequency ultrasound in the 0.2‐1.0 MHz range is commonly used because it is compatible with safety constraints and enables deep tissue penetration. Focused ultrasound (FUS) allows noninvasive access to deep brain regions while maintaining controllable spatial confinement [63]. As a result, ultrasound has also become a promising modality for noninvasive brain stimulation: like magnetic fields, ultrasonic waves can reach deep brain regions without surgery and can be externally focused to target specific areas. FUS already has clinical uses, such as tissue ablation or transient BBB opening, whereas low‐intensity focused ultrasound (LIFUS) can modulate neural activity without ablation [64]. However, direct transcranial ultrasound faces a trade‐off between penetration and precision: lower‐frequency ultrasound penetrates deeper but stimulates a broader area, whereas high‐frequency ultrasound can be focused tightly but is largely attenuated by the skull and tissue [65]. Moreover, ultrasound's effects on neurons are complex, and significant neuromodulatory effects often require relatively high acoustic intensities. Functional nanomaterials offer a solution by amplifying or converting ultrasound signals at the target site, allowing lower‐intensity or lower‐frequency ultrasound to achieve precise, cell‐level stimulation with the help of nanoparticles [66].

Some nanoparticles are able to locally enhance the mechanical effects of ultrasound. Gas vesicles (GVs), for example, are organic gas‐filled nanostructures that act as nanoscale acoustic resonators. When ultrasound passes through tissue, these vesicles oscillate and amplify the mechanical vibration in their vicinity [67]. Hou et al. demonstrated that local injection of GVs into a targeted mouse brain region lowered the ultrasound threshold necessary to evoke robust neuronal activation in that area [65], decreasing the threshold needed to open mechanosensitive ion channels, and triggering calcium influx in nearby neurons. Importantly, only neurons in the GV‐rich region were activated (as evidenced by localized c‐Fos expression), confirming that the effect was confined to where the nanoparticles were present. Other agents like microbubbles and phase‐change nanodroplets can similarly amplify ultrasound, but GVs offer advantages in size and biocompatibility. By adding such amplifiers, effective neuromodulation can be achieved at lower ultrasound intensities and with finer spatial control than ultrasound alone allows.

Another strategy is represented by the exploitation of piezoelectric nanomaterials that generate an electric charge under mechanical stress, converting ultrasound into an electrical stimulus. For example, barium titanate nanoparticles or zinc oxide nanowires deform under ultrasound and generate local electric fields that can depolarize nearby neurons like a tiny electrode [68, 69]. Initial studies showed that vibrating piezo‐nanostructures can produce measurable electrical signals and even influence neurons in culture [70, 71]. Recently, Zhao et al. developed a core–shell nanoparticle with a barium titanate core and a conductive carbon shell (C@BT) that greatly enhanced acoustoelectric conversion efficiency [72]. In a Parkinson's disease zebrafish model, systemic C@BT delivery followed by pulsed ultrasound increased neuronal calcium signals and upregulated synaptic and dopamine‐related markers, leading to improved motor function in the animals. Control experiments confirmed that neither ultrasound alone nor nanoparticles alone produced such effects, verifying that neuromodulation arose from the acoustic‐to‐electric conversion. This approach parallels the magnetoelectric one but uses sound as energy source. Given the widespread availability of clinical ultrasound, acoustoelectric nanoparticles could enable noninvasive deep‐brain stimulation when delivered to target regions.

In summary, ultrasound‐responsive nanomaterials combine the depth penetration and focusability of ultrasound with the precision of nanoscale effectors [73]. By using nanoparticles to concentrate mechanical effects (as in the case of GVs) or to convert ultrasound into other stimuli (electric or thermal), these approaches overcome the limitations of plain ultrasound neuromodulation [65, 74]. Researchers are also exploring ultrasonic nano‐heaters and sono‐chemical catalysts to further expand ultrasound‐based neuromodulation, for instance, using ultrasound to trigger a targeted drug release in the brain [75]. The field is still maturing, but it holds great promise. Since clinical ultrasound technology and safety protocols are already well established, translating nanoparticle‐assisted ultrasonic neuromodulation to clinical use could be relatively straightforward. The non‐ionizing nature of ultrasound and its ability to reach deep targets noninvasively make it a highly compelling energy source to pair with functional nanomaterials for next‐generation brain interfaces.

## 3 Organic Nanoplatforms: Toward Compatible and Degradable Brain Interfaces

**FIGURE 3:**
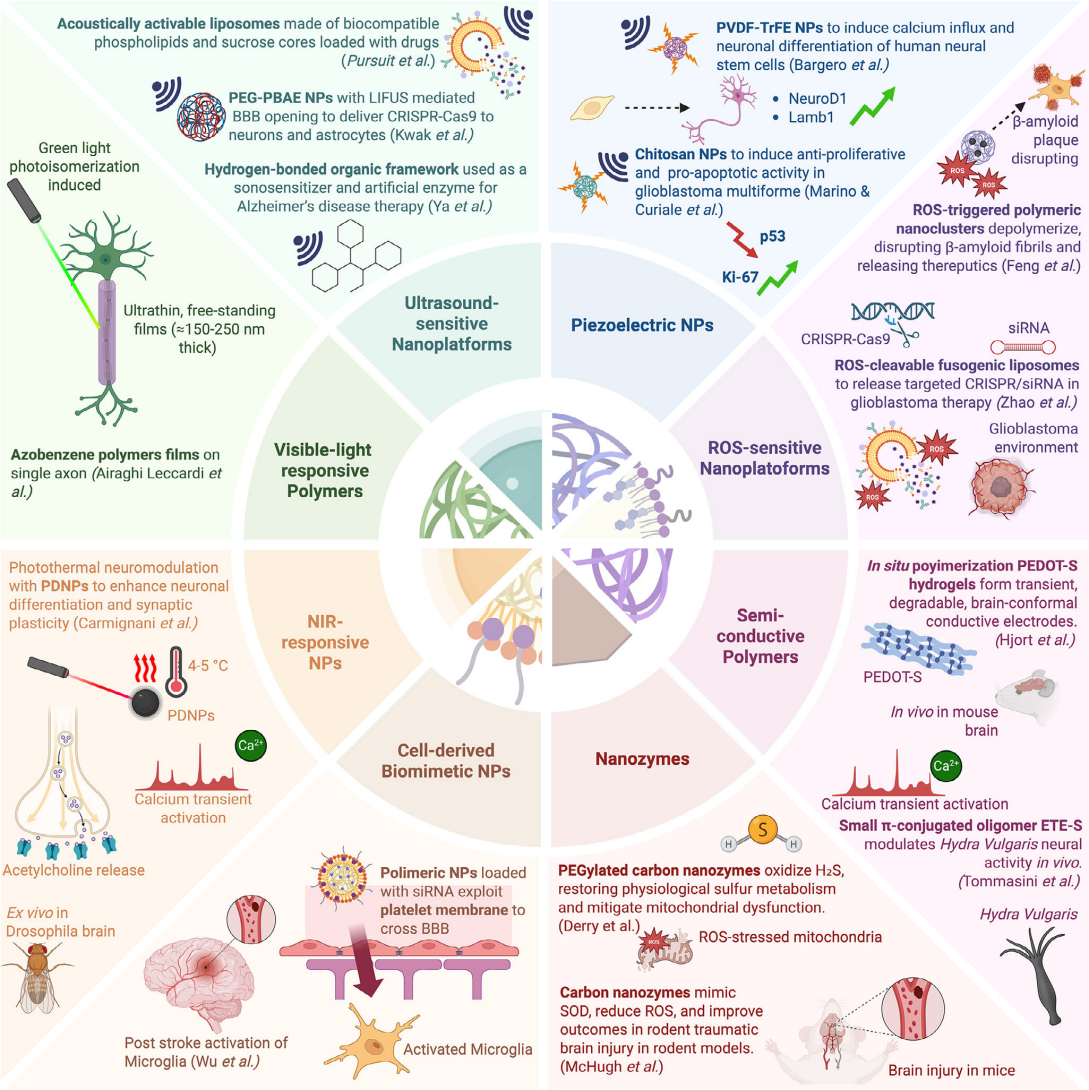
Schematic overview of organic and bio‐derived nanoplatforms for precision brain interfacing. Clockwise from top left: ultrasound‐sensitive nanoplatforms for on‐demand drug and gene delivery; piezoelectric polymer nanoparticles enabling ultrasound‐driven neuronal differentiation or glioblastoma ablation; ROS‐responsive nanoplatforms that depolymerize β‐amyloid or release CRISPR/siRNA in oxidative niches; semiconductive polymers forming in situ PEDOT‐S hydrogels or small π‐conjugated oligomers that directly modulate neural activity; carbon‐based nanozymes that restore redox homeostasis; cell‐derived and biomimetic nanoparticles that cross the BBB and regulate neuroinflammation; NIR‐responsive and visible‐light‐responsive polymers and nanoparticles that mediate photothermal or photomechanical neuromodulation. Together, these examples highlight how organic nanomaterials integrate stimuli‐responsiveness, biodegradability, and biofunctionality to support neuromodulation, gene therapy, and metabolic reprogramming in the brain. Created with BioRender.com.

Recent advances in organic nanomaterials are evolving how brain‐machine interfaces (BMIs) are designed, enabling devices that better match and communicate with neural tissue. Unlike traditional inorganic or metallic implants, organic materials provide biodegradability, ionic‐electronic conductivity, chemical adaptability, and mechanical compliance, properties that enable dynamic communication with cells and tissues. Recent advances have shown how these nanoplatforms can serve as transducers, actuators, and catalysts, translating optical, acoustic, or mechanical energy into bioelectrical and biochemical signals [76]. In this section, we will discuss examples of smart organic nanomaterials that interface with the brain to perform diverse functions, from signal transduction to therapeutic modulation, emphasizing how their chemical versatility, biodegradability, and biomimetic properties offer distinct advantages over inorganic counterparts for developing next‐generation neural interfaces. A schematic overview of these organic and bio‐derived nanoplatforms and their functional roles in precision brain interfacing is shown in Figure 3.

### 3.1 Cell‐Derived Biomimetic Nanoparticles

The BBB remains the foremost obstacle for both drug delivery and nanoscale interfacing with the central nervous system (CNS). Its selective permeability protects neural tissue but severely limits the transport of exogenous molecules and nanoparticles [77]. Organic nanoplatforms, particularly those that incorporate bio‐derived membranes or biomimetic ligands, represent an innovative strategy for traversing this barrier while maintaining biocompatibility [78]. In a pivotal study, Wu et al. engineered biomimetic nanocomplexes that replicate cellular membrane functionality to deliver siRNA to microglia in post‐stroke models [79]. These hybrid nanostructures employ endogenous membrane components to mediate receptor‐driven transcytosis across the BBB, enabling precise gene silencing and modulation of neuroinflammation. Their dual nature, with a synthetic core and biologically derived shell, exemplifies how organic nanomaterials can integrate with physiological systems, using native pathways for transport and signaling [79]. Such biomimetic organic nanocarriers mark an essential step toward BMIs capable of chemical communication across biological barriers, where the interface is not merely tolerated by the brain but recognized as a biological extension of it.

Beyond their use as drug carriers, cell‐derived vesicles can also be used to directly modulate synaptic function. A clear mechanistic example is provided by Vilcaes et al., who isolated extracellular vesicles secreted by hippocampal neurons and showed they carry synaptic‐vesicle‐associated proteins, including the main synaptic vesicle SNARE mediating fusion, synaptobrevin‐2 (VAMP2) [80]. When applied to recipient neurons, these neuronal extracellular vesicles are internalized, and their VAMP2 pool rapidly incorporates into the presynaptic vesicle cycle via a CD81‐dependent process, leading to an increase in inhibitory neurotransmission and even rescuing spontaneous release in synapses lacking endogenous VAMP2. Although this study uses native extracellular vesicles rather than synthetic carriers, it demonstrates that nanoscale, cell‐derived vesicles can transport functional synaptic proteins and reconfigure neural network communication.

### 3.2 NIR‐Responsive Nanoparticles

Beyond passive interfaces, recent advances have focused on organic active nanotransducers, materials capable of converting external physical stimuli into biochemical or electrical signals within neural tissue. These dynamic systems represent the next frontier of BMIs, enabling remote, minimally invasive modulation of neuronal activity through light, ultrasound, or mechanical cues. Light offers optimal spatiotemporal control over neural activation, but its clinical use is constrained by limited tissue penetration and the need for invasive optics. To overcome these issues, organic NIR‐responsive materials have gained attention for deeper, non‐invasive photothermal neuromodulation. Our group developed polydopamine nanoparticles (PDNPs) as fully organic photothermal nanotransducers that absorb NIR light and convert it into localized heat, raising intracellular temperature by ∼4°C–5°C [81]. When exposed to short laser pulses, these nanoparticles trigger calcium transients and acetylcholine release in neuron‐like SH‐SY5Y cells and ex vivo in *Drosophila* brains, demonstrating remote, reversible stimulation. Importantly, PDNPs possess intrinsic antioxidant properties, mitigating oxidative stress associated with thermal activation, a limitation typical of metal‐based photothermal agents. Proteomic analyses further revealed that PDNP exposure enhances neuronal differentiation and synaptic plasticity markers, suggesting their dual function as stimulatory and neurotrophic agents [81]. This study laid the groundwork for bio‐integrated photothermal interfaces capable of modulating neuronal activity without the need for genetic modification.

### 3.3 Visible‐Light‐Responsive Polymers

Complementing NIR stimulation, Airaghi Leccardi et al. introduced a visible‐light‐responsive platform based on azobenzene polymers. These ultrathin free‐standing films (∼150–250 nm thick) undergo photoisomerization‐induced rolling under green light, forming microtubes (0.5–2.0 µm in radius) that conform to neuronal axons and dendrites [82]. By wrapping cellular structures, these polymeric films achieve molecular‐scale contact with the neuronal membrane, a critical feature for high‐fidelity BMIs. In their current form, the films act as purely mechanical actuators, providing light‐triggered, reversible wrapping at cellular resolution without detectable toxicity, and thereby establishing a stable, intimate interface with the neuronal membrane [82]. This approach represents a fully organic, visible‐light‐responsive system that can conform to individual neuronal processes and could be integrated in future designs with conductive or piezoelectric components to enable efficient and wireless control of neural activity at cellular and subcellular scales.

### 3.4 Ultrasound‐Sensitive Nanoplatforms

While optical systems offer precision, their limited penetration depth restricts applications for non‐invasive deep brain stimulations in humans. Ultrasound, conversely, can penetrate several centimeters into tissue and be focused noninvasively, making it ideal for activating embedded organic nanostructures. Purohit et al. introduced acoustically activatable liposomes (AALs) made entirely of biocompatible phospholipids and sucrose cores that respond to LIFUS [83]. Upon exposure, these liposomes release encapsulated drugs, including ketamine and local anesthetics, directly within targeted brain regions, enabling on‐demand, spatially precise neuromodulation without thermal or cavitation damage. The approach demonstrates high translational potential, as all components are pharmaceutically approved, representing a clinically viable route toward ultrasound‐responsive BMIs [83]. In parallel, Kwak et al. employed biodegradable poly(ethylene glycol)‐poly(β‐amino ester) (PEG‐PBAE) nanoparticles in conjunction with focused ultrasound‐mediated BBB opening to deliver mRNA, pDNA, and CRISPR‐Cas9 complexes to neurons and astrocytes [84]. The method achieved in vivo gene editing in defined cortical regions, introducing a paradigm for ultrasound‐gated gene regulation within the brain using completely organic, degradable nanocarriers [84].

Moving from ultrasound‐sensitive organic nanoparticles to proper nanotransducers, Ya et al. developed a hydrogen‐bonded organic framework (HOF) that serves as a sono‐sensitizer and artificial enzyme for Alzheimer's disease therapy [85]. This organic, porphyrin‐based framework incorporating manganese ions generates singlet oxygen and mimics superoxide dismutase and catalase activity under ultrasound exposure, reducing amyloid‐β aggregation and restoring cognitive function in transgenic mice. By combining catalytic and acoustic responsiveness, this biocompatible HOF embodies the ideal multifunctional organic sono‐transducer, capable of both physical stimulation and biochemical response deep within the brain [85].

### 3.5 Piezoelectric Nanoplatforms

In parallel, piezoelectric organic nanotransducers convert mechanical cues, such as LIFUS, into localized electrical signals that modulate neural circuits, providing a minimally invasive pathway toward polymer‐based BMIs [86, 87]. Our group demonstrated that poly(vinylidene fluoride‐trifluoroethylene) (P(VDF‐TrFE)) nanoparticles generate electric potentials upon ultrasound excitation, inducing calcium influx and neuronal differentiation of human neural stem cells [88]. Gene expression analyses revealed upregulation of NeuroD1 and Lamb1, key transcription factors in neurogenesis, confirming that piezoelectric nanostimulation promotes neuronal maturation independently of growth factors [88]. Because these nanotransducers are composed of fully organic polymers, they provide a wireless, electrode‐free approach to neural activation suitable for regenerative applications. Following this direction, we further investigated a piezoelectric nanoplatform based on chitosan, a fully biodegradable and FDA‐approved polymer already used in multiple biomedical devices [89]. Chitosan nanoparticles showed pro‐apoptotic activity in glioblastoma multiforme under ultrasound activation: [89] this finding expands the applicability of organic piezoelectric systems from regenerative to oncological contexts, demonstrating that piezoelectric activation can either promote or inhibit neural activity depending on the microenvironment [87, 89, 90]. Finally, Liang et al. presented a polydopamine‐nanocellulose hydrogel with intrinsic piezoelectric and immunomodulatory properties [91]. When stimulated by ultrasound, the hydrogel generated local electric fields that polarized microglia toward an anti‐inflammatory phenotype and enhanced neural stem cell differentiation in traumatic brain injury models [91]. Together, these studies establish piezoelectric organic nanomaterials as mechanical‐electrical bridges between ultrasound and biological signaling, offering versatile tools for both neurostimulation and neuroprotection.

### 3.6 Organic Nanoplatforms Driven by Endogenous Signals

A defining feature of advanced BMIs is the ability to sense and respond to endogenous biochemical signals without external input. Feng et al. introduced redox‐sensitive polymeric nanoclusters that autonomously respond to the pathological oxidative environment characteristic of Alzheimer's disease [92]. These nanoclusters undergo self‐depolymerization in the presence of ROS, leading to the disassembly of β‐amyloid fibrils and the controlled release of therapeutic agents such as rapamycin and mRNA within activated microglia [92]. By restoring redox equilibrium and enhancing amyloid clearance, this system exemplifies an intelligent organic nanoplatform that couples therapeutic delivery with intrinsic biochemical sensing. Similarly, Hjort et al. pioneered the in situ polymerization of self‐doped poly(3,4‐ethylenedioxythiophene) (PEDOT)‐S derivatives directly within brain tissue, where injected monomeric precursors undergo spontaneous assembly into conductive, ionically permeable hydrogels [93]. These bioresorbable electrodes provide transient electrical coupling to neurons, enabling both recording and stimulation before naturally degrading into non‐toxic byproducts [93]. Their in vivo formation minimizes surgical invasiveness and establishes a foundation for self‐assembling organic bioelectronics capable of adapting to the dynamic neural microenvironment. In parallel, Zhao et al. developed ROS‐cleavable fusogenic liposomes (“Plofsomes”) designed for self‐triggered intracellular delivery of CRISPR‐Cas9 ribonucleoprotein complexes and siRNA in glioblastoma [94]. Upon encountering elevated ROS levels in tumor tissue, a cleavable polymeric “lock” detaches from the liposomal surface, restoring fusogenicity and ensuring precise cytoplasmic release of the genetic cargo [94]. This responsive behavior allows spatiotemporal control of gene editing and silencing without external stimuli, enhancing both therapeutic specificity and biosafety. Tommasini et al. demonstrated that the organic semiconducting oligomer 4‐[2‐{2,5‐bis(2,3‐dihydrothieno[3,4‐b][1,4]dioxin‐5‐yl)thiophen‐3‐yl}ethoxy]butane‐1‐sulfonate (ETE‐S) can directly modulate neural activity in vivo, inducing controlled behavioral responses in the simple nerve net of *Hydra vulgaris* [95]. The conjugated thiophene trimer acted as a molecular neuromodulator, altering calcium‐dependent neuronal signaling and electrical bursting patterns without external stimulation [95]. This discovery reveals that small, π‐conjugated organic oligomers can intrinsically interface with neuronal circuits, providing a chemical pathway for wireless neuromodulation in fully organic brain‐machine systems.

### 3.7 Nanozymes

In addition to externally or internally triggered systems, a parallel line of research is exploring organic nanozymes, nanostructures that mimic enzymatic activity while maintaining the structural stability of synthetic materials. Such systems can act as metabolic stabilizers within the neural milieu, catalyzing redox reactions that preserve homeostasis and protect against oxidative injury [96]. Derry et al. synthesized PEGylated hydrogenated carbon clusters capable of catalyzing the oxidation of hydrogen sulfide into polysulfides and thiosulfate, thus modulating intracellular redox signaling in neuronal and endothelial models [97]. These nanozymes restore physiological sulfur metabolism and mitigate mitochondrial dysfunction, suggesting applications in disorders characterized by oxidative imbalance, such as Down syndrome and neuroinflammation [97]. Building on this, McHugh et al. produced oxidized activated charcoal nanozymes optimized to mimic superoxide dismutase activity [98]. In rodent models of traumatic brain injury, these nanozymes reduced ROS, improved cerebral perfusion, and restored neurological function. Notably, the entirely carbon‐based, PEG‐functionalized architecture provides a fully organic catalytic platform with high stability, tunable surface chemistry, and long in vivo retention [98], making them ideal biochemical mediators for BMIs. Collectively, Derry and McHugh's studies highlight a new paradigm in which catalytic organic nanomaterials act as biochemical transducers, translating oxidative stress signals into restorative redox activity. Unlike conventional electrodes, which record or stimulate electrically, nanozymes operate metabolically, modulating the brain's chemistry to achieve homeostatic balance and enhancing the long‐term compatibility of organic neural interfaces.

Across these advances, organic nanoplatforms emerge as multimodal mediators capable of optical, acoustic, mechanical, and biochemical communication with the brain. They dissolve the traditional distinction between device and tissue, since they can participate in the same physical and chemical signaling processes as neurons themselves. From BBB barrier shuttles to photo or sono‐responsive transducers and catalytic nanozymes, research is focusing on BMIs that prioritize minimal invasiveness and advance toward biodegradable, stimuli‐responsive nanoscale platforms able to integrate and communicate precisely with the living brain.

## 4 Vision Rescue With Nanotransducers

Vision is one of the most sophisticated sensory processes, translating light into complex patterns of neural activity that allow perception, orientation, and interaction with the environment. Major causes of vision loss arise from the degeneration of retinal photoreceptors, typically induced by diseases such as age‐related macular degeneration and retinitis pigmentosa [99]. However, even after photoreceptor loss, the inner retinal neurons remain largely intact despite being disconnected from light input, making them an attractive therapeutic target for vision restoration [100]. Conventional approaches, such as electrical prostheses, gene therapy, and optogenetics, offer partial recovery but are limited by issues in spatial resolution, invasiveness, and long‐term integration [101]. Current retinal prostheses primarily aim to bypass photoreceptors by electrically stimulating the remaining inner retinal neurons, particularly the retinal ganglion cells (RGCs), which maintain functional connections to visual centers in the brain, thereby preserving their ability to convey visual information [102]. However, patients implanted with current‐generation retinal prostheses generally perceive diffuse light spots or phosphenes that lack contrast and spatial definition [103], a consequence of uncontrolled current spread within retinal tissue [104, 105]. Increasing electrode density has not effectively translated into higher visual acuity because the isotropic dispersion of current stimulates large populations of ganglion cells simultaneously, rather than targeting specific cells. Moreover, bypassing intermediate retinal processing layers means that crucial features, such as color, contrast, and motion sensitivity, are lost [103, 106].

Gene therapy and optogenetics have made significant progress by conferring photosensitivity to surviving cells; however, these approaches remain limited by cell‐type targeting efficiency, viral delivery constraints, and the requirement for high‐intensity or narrow‐band light stimulation [107]. Recent advances in optical neuromodulation capitalize on the eye's intrinsic transparency to achieve precise, non‐contact stimulation of retinal neurons [108]. However, the need to balance efficient light delivery with tissue safety and wavelength‐dependent absorption has motivated exploration of hybrid or alternative energy transduction mechanisms that can operate deeper within the retina without invasive interfaces. Balancing light penetration, safety, and wavelength‐dependent absorption remains challenging, motivating the exploration of hybrid nanotechnologies that can transduce light into localized bioelectrical signals within the retina without the need for invasive interfaces.

Unlike conventional prosthetic arrays that rely on macroscopic electrodes, nanotransducers operate at the cellular or even subcellular scale, converting light into localized electrical, thermal, or mechanical cues that directly modulate neuronal excitability. Engineered from diverse materials, including semiconducting polymers, conjugated organic molecules, metallic nanorods, and hybrid peptide scaffolds, these nanoscale systems can be delivered through minimally invasive routes and externally activated with patterned light, enabling wireless and spatially precise modulation of retinal circuits. Building on these principles, the following sections explore the main classes of nanotransducers developed for vision restoration, describing their mechanisms of light‐driven neural activation, representative preclinical demonstrations, and translational challenges.

### 4.1 Photovoltaic Nanoparticles Based on Semiconducting Materials

**FIGURE 4:**
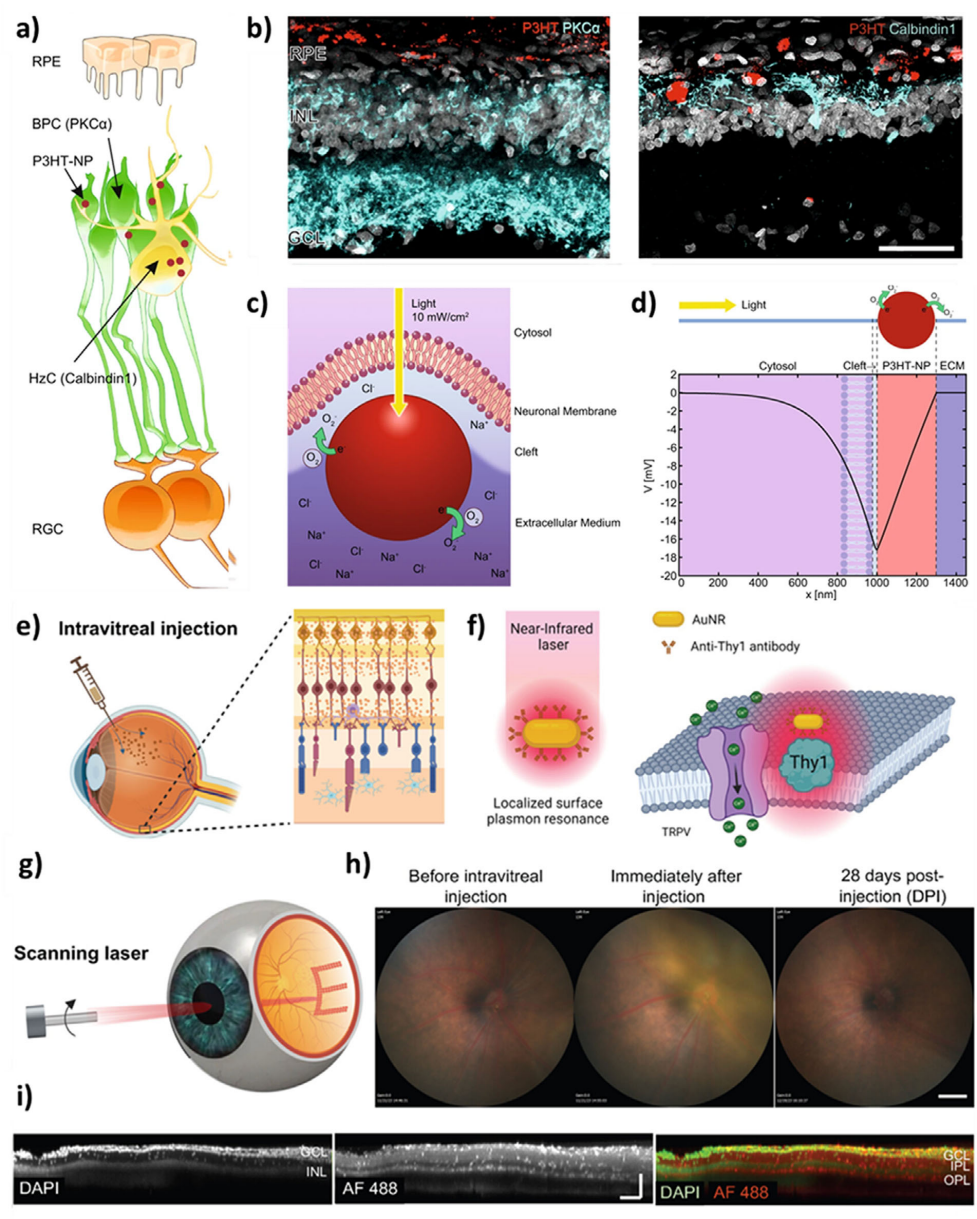
Nanoparticle‐based strategies for vision restoration. (a‐d) Photovoltaic nanoparticles: (a) Schematic of subretinally delivered P3HT‐NPs (red) forming hybrid contacts with PKCα‐positive bipolar cells (rBPCs) and calbindin1‐positive horizontal cells (HzCs). (b) Representative retinal sections from 13‐15‐month‐old RCS rats showing P3HT‐NPs (red) and labeled rBPCs or HzCs (blue) within the retinal pigmented epithelium (RPE), inner nuclear layer (INL), and ganglion cell layer (GCL) (scale bar: 50 µm). (c) Schematic of the nanoparticle microenvironment, including the cleft contacting second‐order neurons. (d) Modeled electric potential across the computational domain comprising the cleft, nanoparticle, and extracellular medium. Light reaches the NPs from the cleft side, where neuronal coupling occurs. Adapted with permission from [113], 2022, Nature Group. (e–i) Plasmonic nanoparticles: (e) Schematic of intravitreally delivered AuNRs in degenerated retina. (f) Mechanism of NIR‐driven photothermal neuromodulation: AuNRs bound to Thy1‐positive neurons via anti‐Thy1 antibodies activate TRPV channels upon plasmonic heating. (g) Scanning NIR laser delivering patterned stimulation. (h) Fundus images before, immediately after, and 28 days post‐injection (DPI). (i) Maximum‐intensity projection showing DAPI‐stained nuclei, Alexa Fluor 488‐labeled AuNRs, and merged channels. Adapted with permission from [115], 2025, ACS.

Among the most advanced nanotransduction strategies for vision restoration, we find organic semiconducting materials that act as artificial photoreceptors, capable of converting light into local electrical stimuli that reactivate residual retinal circuits (representative examples are reported in Figure 4) [109, 110]. These systems exploit the intrinsic photovoltaic and photocapacitive properties of conjugated polymers such as poly(3‐hexylthiophene) (P3HT) and poly[2,6‐(4,4‐bis‐(2‐ethylhexyl)‐4H‐cyclopenta[2,1‐b;3,4‐b′]dithiophene)‐alt‐4,7(2,1,3‐benzothiadiazole)] (PCPDTBT). Their π‐conjugated backbones enable delocalized electron transport and strong optical absorption across the visible spectrum; upon illumination, these polymers generate excitons that can dissociate into free charge carriers at donor‐acceptor interfaces or energy gradients within the material. The resulting separation of charges establishes local electric fields and transient surface potentials that can modulate the membrane potential of adjacent neurons through capacitive coupling. In this way, light is transduced directly into an electrophysiological response, reproducing the graded potentials of native photoreceptors without external bias or wiring. The pioneering study by Maya‐Vetencourt et al. provided the first in vivo evidence of this mechanism using a fully organic retinal prosthesis composed of a P3HT:PEDOT:PSS bilayer supported by a silk fibroin substrate, implanted subretinally in Royal College of Surgeons (RCS) rats, a standard model of retinitis pigmentosa [111]. Optical stimulation of the polymer film elicited retinal and cortical responses and restored visually guided behavior, demonstrating the feasibility of a wire‐free organic photovoltaic interface for sight restoration. The device showed excellent biocompatibility and flexibility, integrating with retinal tissue and maintaining function for months, but its limited spatial coverage and placement sensitivity restricted the extent of recovered vision. Building upon this foundation, Maya‐Vetencourt et al. developed a fully injectable system by replacing the planar film with semiconducting polymer nanoparticles (P3HT‐NPs) [112]. A single subretinal injection allowed the nanoparticles to distribute throughout the subretinal space and establish intimate contact with surviving retinal neurons. These nanoscopic phototransducers reinstated light sensitivity and visually driven behaviors without genetic modification or external electrodes. Although the spatial resolution of P3HT‐NPs has not yet been directly quantified, the grating acuity achieved in dystrophic RCS rats equaled the best values obtained with current retinal implants. This approach dramatically reduced surgical invasiveness while achieving broad retinal coverage, offering the potential to restore the entire visual field through a minimally invasive, wireless nanotransducer system. In a subsequent advance, Francia et al. confirmed that conjugated polymer nanoparticles can restore cortical visual activity and behavior even in advanced‐stage retinitis pigmentosa, where photoreceptors are absent and inner‐retina remodeling is extensive (Figure 4a–d) [113]. In 10‐month‐old RCS rats, subretinally injected P3HT‐NPs restored the pupillary light reflex, visually evoked cortical potentials, and visually guided behaviors to levels comparable to healthy controls. Remarkably, treated animals also regained partial visual acuity and reactivated light‐driven implicit memory traces at multiple cortical levels, with the degree of recovery correlating with the density of nanoparticles and their proximity to second‐order neurons, underscoring the importance of nanoscale coupling at the neuronal membrane. These findings demonstrate that photo‐capacitive stimulation of surviving neurons can overcome severe network remodeling, extending the therapeutic window for intervention in degenerative blindness. Together, these studies demonstrate the feasibility of organic semiconducting nanoparticles as soft, biocompatible interfaces capable of restoring visual responses through local photo‐capacitive stimulation.

### 4.2 Plasmonic and Photothermal Nanotransducers

Another promising class of nanotransducers for vision restoration utilizes plasmonic and photothermal effects to convert NIR light into localized electrical or thermal cues that can stimulate neurons. Metallic nanostructures, particularly AuNRs, exhibit strong surface plasmon resonances in the NIR range, enabling efficient light absorption and scattering with minimal attenuation through ocular media. When illuminated, the collective oscillation of conduction electrons at the nanoparticle surface generates localized temperature gradients and electric field fluctuations, which can depolarize nearby neuronal membranes through thermocapacitive and photothermal mechanisms. Importantly, NIR illumination (700–1100 nm) falls outside the human visual spectrum, allowing activation of retinal neurons without interfering with residual vision in partially sighted individuals. These optical advantages make NIR an attractive spectral window for non‐invasive, wireless neuromodulation, particularly in advanced retinal degeneration where visible‐light photoreception is lost [114]. In a landmark study, Nie et al. demonstrated that intravitreally injected Au nanorods decorated with anti‐Thy1 antibodies (Thy1‐AuNRs) could activate bipolar cells in degenerated retinas through patterned NIR laser illumination, a target traditionally reached through more invasive subretinal injections (Figure 4e–i) [115]. The conjugation promoted selective association with retinal neurons, including RGCs and bipolar cells, the latter forming the critical intermediate layer between photoreceptors and RGCs. Using a custom scanning NIR laser system, the authors delivered dynamic stimulation patterns that selectively triggered ON‐bipolar‐cell activity, as confirmed by calcium imaging and electrophysiological recordings. Notably, bipolar cells were preferentially activated due to the higher thermal sensitivity of their TRPV1 ion channels, which strongly respond to small, rapid temperature increases near physiological ranges. The intravitreal delivery route ensured widespread nanoparticle distribution across the retina, maintaining this effect for several months without systemic toxicity, and establishing a non‐genetic, minimally invasive approach to reactivating degenerated retinal circuits. Mechanistically, Au nanorods function as nanoscale photothermal transducers: briefly, high‐gradient NIR pulses induce rapid, transient heating of the surrounding microenvironment, generating capacitive currents that depolarize neuronal membranes. Depending on the temporal profile and intensity of illumination, the resulting temperature kinetics can be tuned to produce either neural excitation or inhibition, a feature further explored in the work of Begeng et al. [116]. Here, AuNRs engineered for NIR absorption were used to modulate RGC activity through nanoparticle‐enhanced infrared neural modulation in *ex vivo* rat retinas. By varying the pulse duration (100 µs to 200 ms), the authors observed that short pulses generated rapid membrane‐capacitance shifts, leading to depolarization, whereas longer pulses caused sustained heating that suppressed neuronal firing through a thermal block mechanism. This bidirectional control arises from the interplay between the rate of temperature change and the absolute temperature increase, with the former driving excitation and the latter inhibition. Such dynamic modulation represents a conceptual step toward smart nanotransducers capable of adaptive, feedback‐controlled neural interfacing. Beyond metallic plasmonic systems, Wang et al. recently reported a broadband semiconducting nanotransducer based on tellurium nanowire networks (TeNWNs) that extends light sensitivity from the visible into the NIR‐II range (up to 1550 nm) [117]. These nanowires possess a narrow bandgap (∼0.3 eV) and intrinsic structural asymmetries that enable efficient, bias‐free photocurrent generation with current densities exceeding 30 A cm^−2^, several orders of magnitude higher than conventional polymeric devices. Subretinal implantation in blind mice restored pupillary reflexes, cortical visual activity, and visually guided behaviors under both visible and NIR light, while nonhuman primates exhibited robust retina‐derived responses and newly acquired infrared perception without compromising normal vision. Unlike organic polymer nanoparticles, which are limited to the visible spectrum, or metallic nanorods relying on heat‐based stimulation, TeNWNs directly transduce broadband photon energy into electrical activity, combining the high sensitivity of semiconductors with the extended spectral reach of plasmonic systems. This breakthrough demonstrates the feasibility of bias‐free, multispectral retinal prostheses that can both restore vision and augment sensory perception. Collectively, plasmonic and broadband nanotransducers expand the operational spectrum of optical neuromodulation from visible to deep NIR wavelengths. Their non‐contact, wavelength‐tunable activation enables the targeted stimulation of specific retinal layers while minimizing interference with residual vision and the need for invasive surgery, thereby defining a versatile platform for next‐generation, wireless artificial photoreception.

The studies here reviewed collectively highlight the potential of nanotransducer‐based retinal interfaces as a transformative paradigm for vision restoration. A central consideration for their translation lies in the mode of delivery. Subretinal injection, used for organic semiconducting nanoparticles (P3HT‐based) and TeNWNs, allows intimate contact with second‐order neurons, resulting in strong capacitive coupling and widespread coverage of the subretinal space, as demonstrated for P3HT‐NPs that restored cortical and behavioral visual responses even in advanced stages of retinal degeneration [112, 113, 117]. However, this route remains a delicate surgical procedure that carries inherent risks, such as retinal detachment, and is limited to the retinal area that can be safely reached in a single injection [101]. In contrast, intravitreal delivery, as used for plasmonic AuNRs, offers a far less invasive alternative that enables repeated or adjustable dosing, prolonged intravitreal residence, and broad retinal distribution while avoiding subretinal surgery [115]. Yet, the challenge remains to ensure efficient diffusion and precise localization of nanoparticles within specific retinal layers, which is critical for targeted stimulation and long‐term safety [101].

From a translational standpoint, both strategies have been validated primarily in rodent models, with limited data available in larger mammals. The tellurium nanowire prosthesis represents a significant advancement, demonstrating robust functional recovery and safety in non‐human primates [117]. Nevertheless, comprehensive studies are still required to assess pharmacokinetics, clearance, immune response, and optical safety in the larger and more complex human eye [101]. Visible‐light‐driven P3HT‐NPs may interfere with residual photoreceptor activity in patients with partial vision, whereas NIR stimulation with AuNRs or Te nanowires avoids this issue and benefits from deeper tissue penetration and reduced scattering, though it requires careful thermal management to prevent local overheating [101, 113].

Material composition further influences long‐term performance: organic semiconducting P3HT‐NPs exhibit excellent biocompatibility and mechanical compliance, integrating seamlessly with neural tissue; however, they may potentially undergo photobleaching or oxidative degradation under chronic illumination [112, 113]. AuNRs, conversely, offer exceptional chemical and optical stability but may accumulate over time due to their limited biodegradability, raising concerns about long‐term clearance and potential microglial activation [101, 115]. Tellurium nanowires occupy an intermediate ground, being both semiconducting and inorganic, offering high efficiency and durability, but with still‐unknown biodegradation and clearance dynamics [117].

Spectral operation also determines functional compatibility: visible‐light‐driven polymer systems risk interference with remaining photoreceptors, while NIR and NIR‐II activation (AuNRs, TeNWNs) circumvent this limitation, enabling stimulation deeper in the retina and across opaque ocular media [114, 117]. However, thermal safety thresholds for NIR‐based photothermal stimulation must be rigorously characterized to avoid protein denaturation or microvascular effects, as highlighted by recent reviews [101].

Ultimately, these complementary technologies delineate a continuum of nanotransducer‐based strategies, ranging from soft organic photovoltaics that mimic native phototransduction to plasmonic nanorods enabling tunable thermal neuromodulation, and to broadband semiconductors that extend artificial photoreception into the infrared. The next phase of development will involve the integration of these modalities with adaptive optical control systems, refining nanoparticle targeting and stability, and expanding studies to large‐animal and preclinical human models to assess visual acuity, durability, and immune compatibility over clinically relevant time scales [101]. The rational combination of material properties, delivery strategy, and optical activation wavelength will determine the trajectory from experimental proof of concept to clinical application, ultimately bringing the prospect of wireless, high‐resolution, and adaptive artificial vision closer to clinical reality.

## 5 Nanotechnologies Empowering Genetically Targeted Neuromodulation

**FIGURE 5:**
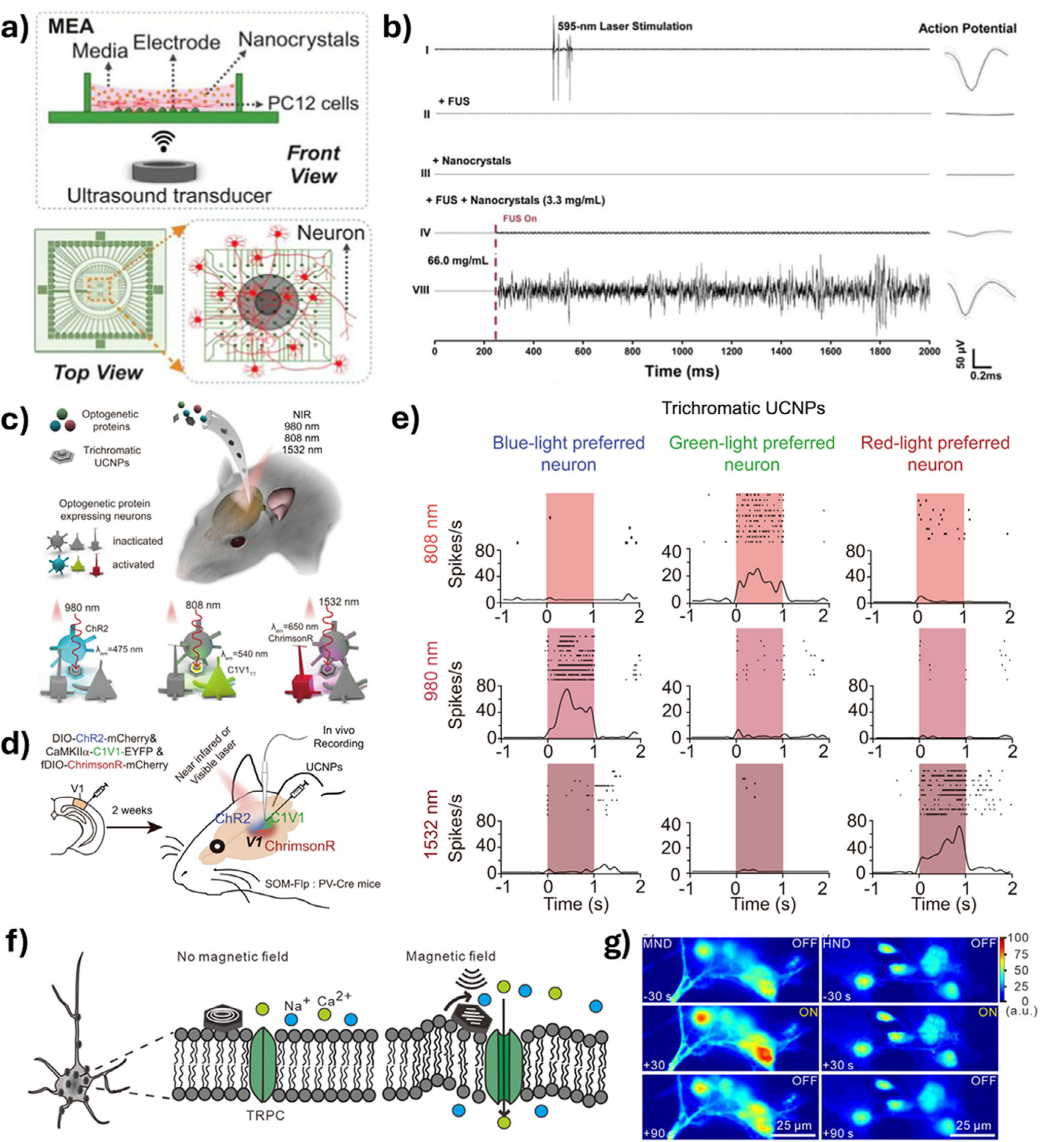
Nanotransducers for genetically targeted neuromodulation. (a, b) Mechanoluminescent nanotransducers for sono‐optogenetics. Schematic representation of an optogenetic neuromodulation setup (a) and the electrical signals recorded by the micro‐electrodes array (MEA) after applying FUS on neuronal cells in the absence and presence of ZnS:Mn^2+^ nanocrystals at different concentrations (b). (c–e) NIR‐driven upconversion nanotransducers for optogenetics. Schematic of NIR trichromatic upconversion manipulated multicolor optogenetics and electrophysiological recordings in mice brain (c, d) and optogenetic responses in blue‐light preferred, green‐light preferred, and red‐light preferred neurons stimulated by NIR light with trichromatic upconversion nanoparticles (UCNPs) (e). (f, g) Nanoparticle‐enabled magnetogenetics. Schematic of magnetomechanical stimulation by using magnetite (Fe_3_O_4_) nanodiscs (MNDs) (f) and color maps of fluorescence intensity of neurons with MNDs compared to non‐magnetic hematite (α‐Fe_2_O_3_) nanodiscs (HND) (g). Adapted with permissions from [22, 119], 2025, ACS & 2021, Nature Group, and [132], 2022, Nature Group.

The convergence of nanotechnology with established genetically targeted neurotechnologies represents an important turning point in the development of precision brain interfacing. Among these, optogenetics stands as a key node in an expanding network of modalities that includes electrophysiology, photonics, chemogenetics, ultrasound neuromodulation, and molecular recording technologies [6]. The synergy is not merely technical but conceptual. Established neurotechnologies, including optogenetics, face well‐known trade‐offs: specificity versus scalability, precision versus chronic stability, and invasiveness versus resolution. Smart nanomaterials are beginning to dissolve these dichotomies by combining optical, electrical, and biochemical domains within an integrated operational platform. This convergence finds expression in stimulus‐responsive nanoplatforms, where nanomaterial functionality is harnessed to wirelessly actuate optogenetic, magnetogenetic, and chemogenetic circuits (Table 1; Figure 5). Acting as nanoscale optical, mechanical, and chemical switches, these platforms enable remote, minimally invasive modulation of defined neural populations, expanding experimental paradigms while opening avenues toward clinical translation, for instance, in neuromodulatory therapies targeting vision, motor circuits, or seizure foci. This section examines key examples where nanomaterials have extended or redefined the capabilities of optogenetics and related genetically targeted techniques, discussing persistent limitations in translation and safety, and envisioning how future iterations of smart nanosystems might enable emerging new functionalities in neurotechnological platforms.

**TABLE 1:** Summary of nanoparticle‐assisted neuromodulation strategies targeting genetically defined neural circuits.

Stimulation Modality	Trigger	Material	Transduction	Genetic Target	Refs.
Sono‐optogenetics (Mechano‐luminescence)	FUS	ZnS:Mn^2^^+^ nanocrystals; Pr^3^^+^‐doped calcium niobates	Mechanical stress into persistent light emission	Opsins (e.g., ChrimsonR)	[119]
Upconversion optogenetics	NIR lights (980‐1532 nm)	UCNPs	NIR into visible light (upconversion)	Multiple opsins	[22, 124]
Magnetogenetics (Magnetothermal)	AMF	Magnetic nanostructures (*e.g*., SPIONs)	Magnetic into localized nanoscale heating (Néel/Brownian relaxation)	Thermosensitive ion channels (*e.g*., TRPV1)	[126, 127, 128, 131]
Magnetogenetics (Magnetomechanical)	Rotating or gradient magnetic fields	Magnetic nanostructures (*e.g*., nanodiscs, anisotropic nanoparticles)	Magnetic into nanoscale torque/force generation	Mechanosensitive channels (Piezo1, TRPC, TRPV4)	[45, 129, 130, 132]
Sono‐Chemogenetics (Ultrasound‐Triggered Ligand Release)	FUS	HOF nanocrystals encapsulating CNO or similar ligands	Ultrasound into ligand release	DREADDs (hM3D(Gq))	[134]
Hybrid and Future Adaptive Nanoplatforms	Multimodal external and physiological cues	Bio‐hybrid nanosystems integrating sensing and actuation	Bidirectional conversion; electrocatalysis; adaptive neuromodulation	Engineered synthetic circuits	[136, 137, 138]

### 5.1 Mechanoluminescent Nanotransducers for Sono‐Optogenetics

Among the emerging classes of smart stimuli‐responsive nanomaterials, mechanoluminescent systems represent a particularly elegant strategy for remote optical control of genetically defined neurons [118]. Mechanoluminescent nanotransducers can convert mechanical energy, typically delivered from FUS, into localized light emission and wirelessly actuate optogenetic circuits deep within tissue. These nanomaterials, therefore, circumvent the need for implanting invasive optical fibers in the brain. A recent example by Wang et al. (Figure 5a,b) [119] demonstrates how defect and size engineering can advance this concept. Through an emulsion‐assisted self‐assembly method with subsequent calcination, the authors synthesized ZnS:Mn^2^^+^ nanocrystals (30–300 nm) whose abundant stacking faults enhanced mechanoluminescent responses. Under low‐pressure ultrasound, these nanoparticles generated bright, persistent light capable of activating optogenetically sensitized neurons (i.e., ChrimsonR‐transduced neuronal PC12 cells), thus functioning as wireless sono‐optogenetic transducers. While further validation in complex in vivo models is needed, this work demonstrates how material design at the nanoscale can enhance the performance and versatility of wireless optogenetic interfaces. At the same time, the persistence of light emission extends stimulation windows but reduces temporal precision. Future developments may exploit the tunability of mechanoluminescent materials, for instance, by adjusting dopant composition to have precise control over emission lifetime [120]. Another crucial direction involves achieving recoverable and nondestructive mechanoluminescence, as demonstrated in Pr^3+^‐doped calcium niobates [121] and ZnS:Mn^2+^ nanocrystals [119], which exhibit repetitive and fatigue‐resistant light emission under mechanical stress. Such characteristics are particularly valuable for chronic optogenetic stimulation, where material stability and reproducibility are fundamental.

Beyond inorganic systems, the emergence of organic mechanoluminescent nanoplatforms introduces new possibilities for biocompatible and biodegradable neural interfaces [122]. Recently, HOFs have been engineered as sono‐sensitized mechanoluminescent nanotransducers, achieving fully metal‐free and highly porous architectures with high biocompatibility [123]. These systems couple ultrasound‐triggered ROS generation with chemiluminescent reactions to deliver light deep within the brain, enabling cell‐type‐specific activation and long‐term recovery of motor function in Parkinsonian rat models.

### 5.2 NIR‐Driven Upconversion and Hybrid Nanotransducers for Optogenetics

While ultrasound‐mediated mechanoluminescence enables deep, wireless photon delivery for optogenetic‐based neuromodulation, NIR light offers complementary advantages for high‐precision studies of the brain of small mammals (i.e., optoneurophysiology). Unlike ultrasound, NIR light cannot penetrate tissues deeply, but in rodents, it provides improved transcranial access with respect to visible light. When combined with UCNPs, NIR light can be converted into specific visible wavelengths to precisely activate multiple optogenetic proteins with minimal tissue heating and reduced nonspecific interactions. This allows for cell‐type‐specific, multicolor modulation of neural circuits, as demonstrated by Liu X. et al. (Figure 5c–e) [22], who independently controlled three neuronal populations in the mouse cortex using 980, 808, and 1532 nm irradiation and finely modulated the motion behavior of awake mice. Towards therapeutic applications, Li et al. [124]. developed a minimally invasive upconversion optogenetic strategy with UCNPs converting transcranial NIR light into blue light to activate channelrhodopsin‐2‐expressing neurons in the globus pallidus of Parkinsonian mice without implanting optical fibers. This method restored motor performance and coordination in behavioral assays, while minimizing inflammation and tissue disruption compared to conventional fiber‐based optogenetics. Expanding this concept toward a genetic modification‐free approach, Jin S. et al. [24]. were able to stimulate deep‐brain regions in wild‐type mice by designing hybrid upconversion‐photovoltaic nanoparticles, which combine rare‐earth‐doped UCNPs and WO3‐x photovoltaic nanorods. These nanotransducers convert deeply penetrating NIR light into localized electrical stimuli, eliminating the need for genetic modification and enabling immediate neuronal activation. In vitro patch‐clamp experiments confirmed robust excitation of neurons in brain slices, while in vivo experiments demonstrated that hybrid upconversion‐photovoltaic‐mediated NIR stimulation effectively modulated neuronal activity in deep brain regions, including the medial septum (MS) and ventral tegmental area (VTA). Functionally, transcranial NIR stimulation in the MS suppressed seizure activity and synchronized hippocampal theta oscillations, whereas stimulation in the VTA triggered dopamine release and produced spatial preference behavior in freely moving mice, highlighting the versatility and impact of this approach. However, the elimination of genetic modification, while broadening clinical translatability, inherently reduces cell‐type specificity, shifting the paradigm from targeted population control toward circuit‐level or regional modulation. Moreover, it is important to note that translation of deep‐brain stimulation to humans remains challenging when relying on NIR light, as the globus pallidus lies 5–7 cm beneath the scalp, whereas NIR penetration in brain tissue is limited to only a few millimeters to roughly 1 cm [125].

### 5.3 Nanoparticle‐Enabled Magnetogenetics

In parallel with NIR and mechanoluminescent approaches, nanoparticle‐enabled magnetogenetics is rapidly maturing from a set of proof‐of‐concept demonstrations into a versatile toolkit for wireless, deep‐tissue neuromodulation. Specifically, magnetogenetics exploits magnetic nanoparticles to remotely activate genetically defined neuronal populations through local transduction of magnetic energy into biological signals [126]. In this approach, neurons are engineered to express ion channels or receptors that respond to changes in temperature or mechanical stress, such as the thermosensitive transient receptor potential vanilloid (TRPV) family [127] or the mechanosensitive Piezo channels [128]. When exposed to AMFs or static gradients, magnetic nanoparticles localized near these channels convert magnetic energy into localized heating or nanoscale mechanical forces, thereby triggering channel opening and eliciting neuronal depolarization. Typically, SPIONs or composite magnetic nanostructures are functionalized to target neuronal membranes, vesicles, or specific molecular complexes. Upon exposure to an AMF in the hundreds of kHz range, these particles dissipate energy through Néel and Brownian relaxation losses, raising the local temperature by several degrees in a confined nanoscale volume. When coupled to temperature‐sensitive ion channels like TRPV1, this local heating is sufficient to depolarize neurons with millisecond‐to‐second temporal dynamics [127]. Alternatively, magnetic nanoparticles can be designed to exert piconewton‐scale mechanical forces on mechanosensitive proteins (e.g., PIEZOs and TRPV4) under oscillating or gradient fields, offering a nonthermal pathway for channel activation [129, 130].

This magnetothermal or magnetomechanical transduction enables non‐invasive, deep‐tissue control of neuronal activity, overcoming the limited penetration of visible light through biological tissue. Because magnetic fields can penetrate deep into the brain with minimal attenuation and without generating significant aspecific heating in bulk tissue, magnetogenetics allows for local and remote modulation of neurons without optical fibers or implanted emitters. In animal models, these approaches have been successfully used to activate or inhibit specific brain circuits, evoke behavioral responses, and modulate physiological processes in awake, freely moving mice [131].

A particularly interesting demonstration of this neurotechnique was provided by the introduction of the m‐Torquer system, a magnetomechanical toolkit that delivers piconewton‐scale torques to mechanosensitive ion channels from distances up to 70 cm [45]. The approach couples anisotropic octahedral magnetic nanoparticles to a spherical polymer scaffold, yielding assemblies with a magnetic moment 470‐fold higher than single nanoparticles, while maintaining weak ferromagnetism and long‐term colloidal stability. These nanoscale “torquers” experience Brownian rotation under a rotating, uniform magnetic field generated by a circular magnet array, exerting torque forces of 2–10 pN for activating Piezo1‐expressing neurons. Specifically, neurons were genetically modified to express Myc‐tagged Piezo1 to achieve selective binding of m‐Torquers via anti‐Myc conjugation. The magnetomechanical stimulation at 0.5 Hz triggered robust calcium influx and c‐Fos expression in > 80% of Piezo1‐expressing neurons, with no evidence of membrane disruption or thermal artifacts. Interestingly, in vivo application of m‐Torquers to the motor cortex of freely moving mice induced reproducible locomotor responses and demonstrated precise, reversible modulation of cortical circuits without physical contact or invasive implants. The system also enabled deep‐brain stimulation of the hypothalamus through magnetically driven torque, validating the feasibility of remote mechanogenetic control in subcortical regions. With its unique combination of long‐range actuation, subcellular mechanical specificity, and compatibility with mechanosensitive ion channels, m‐Torquer represents a paradigm shift from heat‐based magnetothermal methods toward direct mechanical neuromodulation. This advance not only addresses key physical limitations of earlier magnetogenetic designs based on magnetothermal stimulation (e.g., slow activation, potential tissue damage, and uncontrolled biological responses), but also lays the groundwork for next‐generation non‐invasive brain interfaces scalable to large animals and potentially humans. More recently, Su et al. [132]. showed that magnetomechanical nanodiscs can activate endogenous TRPC mechanotransduction pathways to achieve wireless neuronal stimulation and untethered deep‐brain modulation in vivo, suggesting a route to transgene‐free magnetomechanical neuromodulation (Figure 5f).

### 5.4 Nanoparticle‐Assisted Chemogenetics and Sono‐Chemogenetics

Nanoparticle‐assisted chemogenetics is an emerging neuromodulation strategy that integrates the cell‐type specificity of chemogenetic receptors (e.g., designer G protein‐coupled receptors such as DREADDs) [133] with the spatiotemporal precision of nanomaterial‐based drug delivery systems. In this approach, nanoparticles are engineered to encapsulate and release chemogenetic ligands in response to remote physical stimuli such as ultrasound, magnetic fields, or light. By converting physical energy into chemical signaling events at precise locations and times, nanoparticle‐assisted chemogenetics allows for selective control of defined neural circuits without direct implantation or continuous systemic drug exposure. This neurotechnique complements magnetogenetic‐based approaches: while magnetogenetics directly couples physical energy to membrane channels or mechanosensitive proteins, nanoparticle‐mediated chemogenetics introduces an intermediate chemical transduction step. This distinction offers several advantages, particularly higher biochemical selectivity and compatibility with established pharmacological pathways. At the same time, it circumvents some limitations of magnetogenetics, such as the limited control over intracellular signaling cascades. A striking implementation of this approach was recently presented by Wang and colleagues [134], who developed a sono‐chemogenetic platform for minimally invasive, genetically targeted deep‐brain stimulation. The authors engineered HOF nanocrystals capable of encapsulating and releasing clozapine N‐oxide (CNO), which is the canonical DREADD ligand, upon FUS stimulation. Interestingly, the ultrasound threshold for activation could be precisely programmed by tuning the density of hydrogen bonds and π–π stacking within the HOF architecture. This design enabled ultrasound‐triggered drug release with sub‐second precision and negligible leakage in the absence of stimulation. In cultured neurons expressing hM3D(Gq) receptors, the TATB@CNO nanocrystals triggered >90% activation with a latency of ∼1.6 s, sustaining excitation for nearly 1 min. When tested in vivo in mice and rats, sono‐chemogenetic stimulation of the ventral VTA produced robust calcium transients, activity‐dependent c‐Fos expression, and reproducible modulation of reward‐related behaviors. Importantly, deep‐brain targeting up to 9 mm below the cortical surface was achieved without tissue damage, glial activation, or BBB disruption, therefore highlighting the safety and potential of this approach. This work clearly bridges two scales of control: the physical precision of ultrasound energy and the molecular selectivity of pharmacological signaling. The approach, therefore, addresses invasiveness, immunogenicity, and off‐target activation, which are known to be the key challenges that limit different neuromodulation platforms. However, several translational barriers remain. The current implementation still depends on local viral delivery to express DREADDs and intracranial injection of nanocrystals, which complicate scalability and translatability. Moreover, while ultrasound‐triggered release improves the timing of drug action, GPCR‐mediated signaling remains inherently slower than direct ion channel modulation, with effects lasting seconds to minutes. Nonetheless, sono‐chemogenetics introduces a paradigm where nanomaterials act not just as physical transducers but as molecular gatekeepers linking mechanical stimuli to biochemical cascades. In the long term, integrating such systems with FUS‐mediated BBB opening could enable noninvasive delivery of both genes and nanoparticles [135], advancing toward fully externalized, precision‐targeted neuromodulation therapies.

Despite the remarkable progress outlined above, the integration of nanotechnology with genetically targeted neuromodulation remains in its early stages of development. Most current implementations still rely on viral transduction, intracranial injections, or animal‐scale field parameters that are not yet compatible with human application, especially those exploiting NIR for deep brain stimulation. Future smart nanosystems should evolve beyond simple stimulus transduction toward adaptive, multifunctional platforms capable of sensing, computation, and feedback [136, 137]. By coupling nanoscale actuation with real‐time molecular readouts, closed‐loop nanoneurotechnologies could dynamically tune neuronal or glial activity based on physiological state, moving from static stimulation to context‐aware neuromodulation. Moreover, the next frontier will likely see the convergence of synthetic biology and materials science, where engineered cells interface seamlessly with intelligent nanomaterials to create hybrid bioelectronic circuits operating within the living brain [138].

## 6 Beyond Neurons: Modulating Glial Cells

**FIGURE 6:**
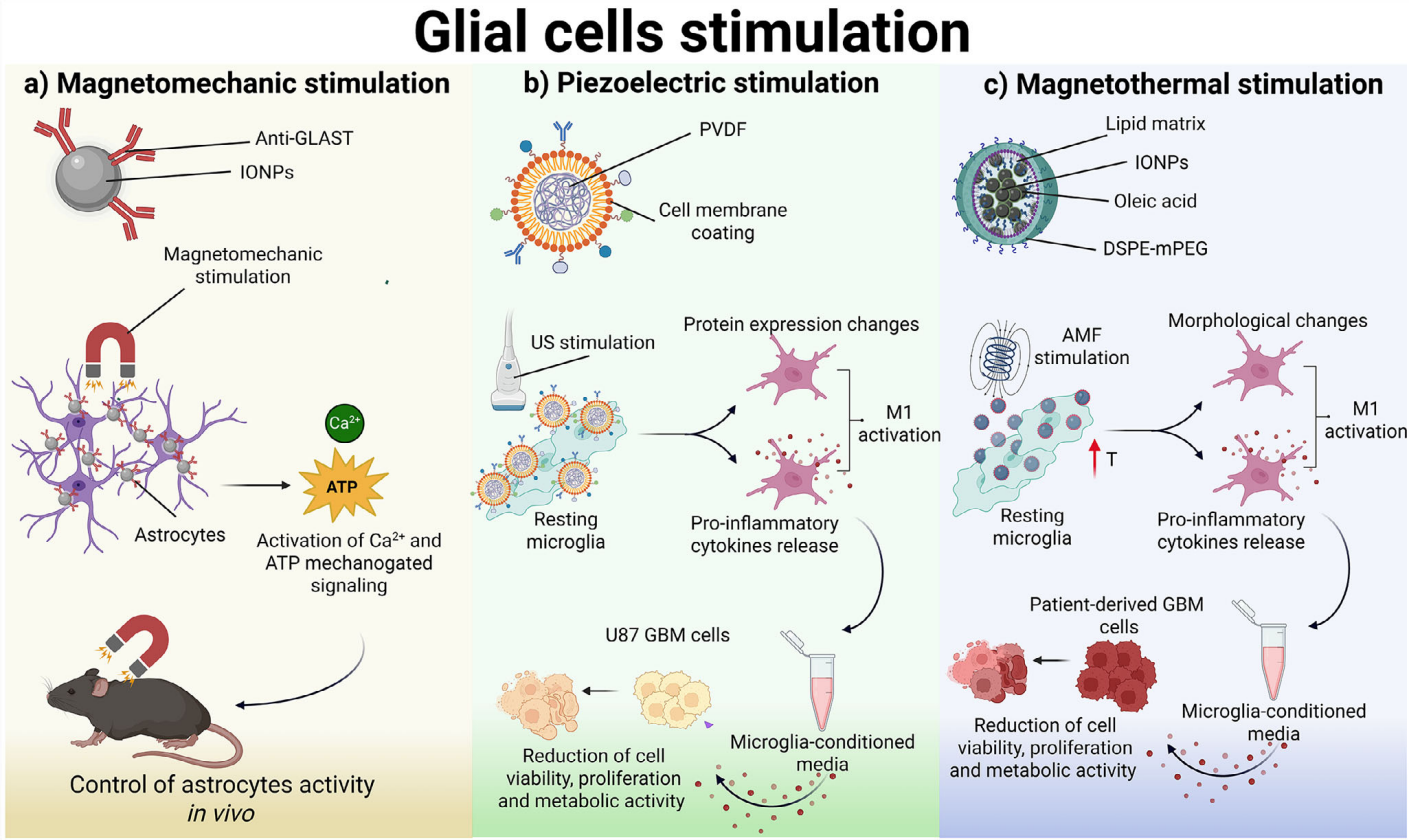
Strategies for remote stimulation of glial cells using nanoparticle‐mediated physical cues. (a) Magnetomechanical stimulation: Magnetic nanoparticles (IONPs) functionalized with anti‐GLAST antibodies target astrocytes. Application of an external magnetic field induces mechanical forces on the particles, activating mechanosensitive signaling pathways, including intracellular Ca^2^^+^ influx and ATP release, enabling remote modulation of astrocyte activity in vivo. (b) Piezoelectric stimulation: Ultrasound (US) activates piezoelectric PVDF nanoparticles coated with cell membranes, generating localized electrical signals that stimulate resting microglia. This triggers changes in protein expression and promotes M1 polarization, leading to the release of pro‐inflammatory cytokines. Microglia‐conditioned media subsequently reduce the viability, proliferation, and metabolic activity of U87 glioblastoma cells. (c) Magnetothermal stimulation: Lipid‐encapsulated IONPs subjected to an alternating magnetic field (AMF) generate localized heating, inducing morphological changes and M1 activation in resting microglia. The resulting secretion of pro‐inflammatory cytokines suppresses viability, proliferation, and metabolic activity of patient‐derived glioblastoma cells. Created with BioRender.com.

For decades, the field of neurotechnology has focused primarily on neurons, the electrical excitable units of the brain, as the principal targets for modulation. Yet, research has fundamentally shifted this neuron‐centric paradigm. Neural computation, plasticity, and even pathology emerge from the dynamic interplay between neurons and the diverse ensemble of non‐neuronal cells that constitute a substantial fraction of the brain's volume [139, 140]. In this scenario, astrocytes and microglia form tightly coupled signaling networks that regulate ionic balance, neurotransmitter turnover, metabolic support, immune surveillance, and neurovascular coupling [141]. Dysregulation in these cell components contributes not only to neurodegenerative pathologies such as Alzheimer's and Parkinson's disease, but also to psychiatric disorders, epilepsy, and chronic pain. From this perspective, precision brain interfacing is evolving “beyond neurons” to engage these non‐excitable yet functionally indispensable cell types. Unlike neurons, glial cells often operate through slow biochemical and Ca^2+^‐based signaling, making them especially amenable to smart material‐mediated biochemical and mechanical modulation. Nanotechnologies provide unprecedented opportunities to sense, interpret, and modulate these signals at cellular and subcellular scales (Figure 6).

Astrocytes represent a critical non‐neuronal target for smart nanotechnologies. Once considered passive support cells, astrocytes are now recognized as active regulators of synaptic function, energy metabolism, and neurovascular coupling, largely through Ca^2+^‐dependent gliotransmission [139]. The ability to modulate astrocytic activity with nanoscale precision opens new opportunities to indirectly tune neural circuits and promote regeneration in disease or injury contexts. However, nanoparticle‐assisted stimulation approaches have been explored primarily in the peripheral nervous system as a strategy to induce Ca^2+^ influx and promote neuronal regeneration through astrocyte‐like glial interfaces [142]. A rare example of nanoparticle‐assisted remote modulation of astrocyte activity in the brain was introduced by Yu et al. [143]. and is based on a genetic manipulation‐free magnetomechanical stimulation approach. Specifically, authors functionalized sub‐micrometer magnetite particles with antibodies targeting the astrocyte‐specific GLAST transporter and exploited the intrinsic mechanosensitivity of astrocytes to trigger controlled Ca^2+^ and ATP signaling via mechanical stress induced by magnetic fields. This technique enabled precise stimulation of astroglial populations in the rat brainstem, producing reproducible cardiovascular responses mediated by astrocyte‐neuron crosstalk. Specifically, the magnetomechanical stimulation of astrocytes within autonomic brainstem nuclei produced reproducible changes in mean arterial pressure and heart rate, consistent with the established role of these astrocytes in central cardiovascular control. Importantly, magnetomechanical stimulation bypassed the need for genetic modification or invasive implants, positioning it as a promising translational route toward clinically viable glial modulation. Beyond its immediate implications, this study delineates a new paradigm in which mechanical rather than electrical or optical signaling is exploited as a primary communication channel with the brain's non‐neuronal cells. The quantitative mapping of astrocytic mechanosensory thresholds (∼0.3 Pa) provides a key biophysical benchmark for designing future magneto‐ or acousto‐mechanical actuators. Despite these advances, the field of astrocyte‐targeted nanomodulation remains in its infancy. The rapid progress in nanomaterial engineering, combined with the growing appreciation of glial heterogeneity and function, suggests that future “smart” nanotechnologies will enable not only selective stimulation but also bidirectional communication with astrocytic and microglial networks, moving into a new era of brain interfacing that transcends neuronal boundaries.

Beyond astrocytes, microglia, the brain's resident immune cells, represent a compelling new target for smart nanomodulation. These cells orchestrate inflammatory responses, synaptic pruning, and neuroprotection, but their phenotype becomes profoundly dysregulated in pathological conditions. In neurodegenerative disorders, microglia frequently adopt a chronically activated, neuroinflammatory M1 state, whereas in brain tumors they often shift toward an immunosuppressive, tumor‐supporting M2 phenotype. The ability to reprogram microglial activity through localized, non‐pharmacological cues would therefore open unprecedented therapeutic avenues for neuroinflammatory and neoplastic disorders.

Among the contributions of our group, we have explored piezoelectric and magnetothermal nanotransducers as tools for remote microglial activation. In a first platform [144], P(VDF‐TrFE) piezoelectric nanoparticles were camouflaged with glioblastoma cell membrane extracts to promote homotypic targeting. When stimulated by low‐intensity ultrasound, these nanoparticles produced localized electric fields at the microglial membrane surface, triggering the polarization of glioma‐associated microglia from an M2 to a pro‐inflammatory M1 phenotype. This reprogramming enhanced the expression of M1 markers and, in co‐culture, significantly reduced the viability and metabolic activity of glioblastoma cells. Remarkably, this work demonstrated for the first time that localized electrical impulses generated at the nanoscale can bias microglial polarization, effectively transforming these innate immune cells into tumor‐suppressive effectors. Complementarily, we also reported a magnetothermal strategy based on lipid‐based magnetic nanovectors incorporating iron oxide nanoparticles [20]. Upon AMF stimulation, lipid‐based magnetic nanovectors induced a controlled rise in intracellular Ca^2+^ levels, leading to M1‐like activation characterized by the upregulation of CD40, CD86, and pro‐inflammatory cytokines (IL‐6, IL‐8, TNF‐α). The conditioned medium from activated microglia exerted cytotoxic effects on both immortalized and patient‐derived glioblastoma cells, confirming a secondary, paracrine anti‐tumor action. Notably, the lipidic matrix enhanced nanoparticle biocompatibility and internalization efficiency [20]. Together, these studies establish a new conceptual direction for glial‐focused precision modulation: rather than suppressing inflammation, nanomaterial‐mediated bioelectrical and magnetothermal cues can be harnessed to therapeutically re‐educate immune cells within the brain's microenvironment.

A summary of the key studies demonstrating nanoparticle‐assisted modulation of astrocytes and microglia is presented in Table 2.

**TABLE 2:** Nanoparticle‐based strategies for the remote modulation of non‐neuronal brain cells.

Target cell type	Modality / trigger	Nanomaterial / platform	Mechanism of action	Biological outcome/Function	Refs.
Astrocytes	Magnetomechanic stimulation	Magnetite particles functionalized with anti‐GLAST antibodies	Activation of mechanosensitive Ca^2^^+^ and ATP signaling	Modulation of cardiovascular responses via brainstem stimulation	[143]
Microglia	Piezoelectric stimulation (ultrasound‐triggered)	Camouflaged P(VDF‐TrFE) piezoelectric nanoparticles	Ultrasound‐induced local electric fields	Polarization shift from M2 (tumor‐supportive) to M1 (pro‐inflammatory) phenotype	[144]
Microglia	AMF	Lipid‐based magnetic nanovectors loaded with SPIONs	AMF‐mediated local heating and Ca^2+^ influx	Induction of M1‐like activation	[20]

## 7 Nanosensors for Precision Brain Interfacing

Understanding brain activity across molecular, cellular, and circuit scales requires tools capable of resolving fast, localized, and physiologically relevant signals. Traditional interfaces, such as microwire electrodes and fluorescent reporters, have driven major advances; however, they remain limited by mechanical mismatch with neural tissue, progressive degradation of the tissue‐device interface, restricted chemical specificity, or limited access to subcellular environments [145, 146]. These limitations can be overcome by exploiting the intrinsic advantages of materials at the nanoscale: high surface‐to‐volume ratios that enhance sensitivity, tunable optoelectronic and electrochemical properties that enable chemical selectivity, and mechanical flexibility that better matches the softness and curvature of the brain [1]. As a result, nanosensors are emerging as a central class of tools for precision brain interfacing, capable of recording electrophysiological, neuromodulatory, and ionic signals with minimal invasiveness and improved chronic stability. This section reviews two major domains of nanosensor development for in vivo brain monitoring, nanoelectronic interfaces designed to stably record electrical activity with subcellular precision, and optical and electrochemical nanosensors engineered to detect neurotransmitters and ions deep within intact neural circuits. Together, these advances demonstrate how nanoscale design principles can transform neural recording technologies across functional modalities (Table 3).

**TABLE 3:** Representative nanosensors for precision brain interfacing.

Nanosensor	Modality/target signals	Key strengths	Main limitations / challenges	Refs.
Syringe‐injectable microporous mesh nanoelectronics	Electrical / local field potentials, action potentials	Minimally invasive, compliant, tissue‐like integration, multiplexed recording	Delivery and packaging complexity, limited translation	[148, 149]
Nanoelectronic thread electrodes	Electrical / action potentials	Subcellular size, ultraflexible, scar‐free integration, stable chronic recording	Shuttle‐assisted implantation, limited scalability, packaging issues	[150]
Ultrathin high‐density electrode arrays	Electrical / action potentials	Ultraflexible, high‐density recording, high signal‐to‐noise ratio	Implantation and packaging complexity, limited long‐term validation	[151]
Nanowire‐based probes	Electrical / local field potentials, action potentials, intracellular signals	Subcellular precision, high sensitivity, intracellular access	Complex implantation, limited in vivo validation, unresolved chronic translation	[152, 153]
SWCNT optical nanosensors	Optical / dopamine, catecholamines	NIR readout, photostable, high spatial resolution, sub‐second kinetics	Delivery/localization issues, limited chronic stability, narrow analyte range	[156, 157]
Carbon nanotube yarn microelectrodes	Electrochemical / serotonin, monoamines	High temporal resolution, antifouling behavior, stable and sensitive response	Limited chronic validation, signal saturation, implantation issues	[158]
UCNP‐based K+ nanosensors	Optical / K+ dynamics	NIR excitation, sensitive ratiometric readout, deep‐tissue compatibility	Optical‐access dependence, limited long‐term stability, calibration/delivery issues	[159]

### 7.1 Nanoelectronic Interfaces for Electrical Recording

Nanoelectronic interfaces exploit nanometer‐scale conductors, transistors, and ultraflexible structures to record electrical activity with high spatial precision and reduced tissue damage. Because brain tissue is soft and structurally complex, mechanical matching between probes and neural tissue is critical for stable, chronic interfaces [147]. Recent developments in syringe‐injectable, ultraflexible, or nanowire‐based electrodes address this challenge by designing devices whose dimensions and stiffness approach those of the surrounding brain. Syringe‐injectable mesh nanoelectronics represent a paradigm shift in chronic neural interfacing. Liu et al. introduced an ultraflexible macroporous mesh that can be delivered directly into brain tissue through a needle, unfolding to form a stable, tissue‐like interface [148]. These devices, composed of nanoscale metal conductive traces embedded in polymer filaments, more closely match the mechanical properties of neural tissue than conventional electrodes. Their three‐dimensional open architecture dimensions allow neurons, axons, and glia to interpenetrate the mesh, enabling seamless structural integration and reducing foreign‐body response, and supporting multiplexed in vivo neural recording. Follow‐up work by Zhou et al. demonstrated the penetration of neurons and neurofilaments into the mesh, as well as long‐term biocompatibility of these systems [149]. Systematic histological studies over weeks to three months showed minimal activation of astrocytes and microglia around implanted meshes, in strong contrast to the dense glial encapsulation observed around traditional probes. These findings highlight how nanoscale flexibility and porosity can be leveraged to achieve chronic integration essential for long‐term brain monitoring. Thread‐like nanoelectronic probes further refine this concept. Luan et al. developed nanoelectronic thread electrodes, which are subcellular‐scale conductive filaments with micrometer‐level cross‐sections and nanometer‐thick encapsulation, that enter the brain with minimal disruption and maintain glial scar‐free integration [150]. Their extreme flexibility substantially reduced micromotion‐induced injury, a major limitation of conventional electrodes, while enabling high‐density electrical recording. Together, mesh and thread‐based architectures illustrate how nanoscale structural design can deeply reshape the chronic stability of brain interfaces.

Complementing these injectable meshes, ultraflexible high‐density electrode arrays have been developed for intracortical recording. Wei et al. fabricated sub‐micrometer‐thick arrays capable of conforming to brain microcurvature and producing stable, high‐channel‐count electrophysiology [151]. Their nanoscale thickness significantly reduces bending stiffness, enabling conformal contact with brain tissue and mitigating micromotion‐induced artifacts. This class of devices provides the scalability needed for population‐level electrophysiology while maintaining the minimal invasiveness required for chronic stability. Nanowire electrodes offer a distinct route toward high‐resolution brain monitoring, with the potential for intracellular or juxtacellular access at subcellular precision. Silicon nanowires can be engineered as field‐effect transistors, where modulation of channel conductance reflects local membrane potentials. Delacour et al. introduced neuron‐gated silicon nanowire field‐effect transistors that record local field potentials and unitary spikes with high sensitivity [152]. Their nanoscale dimensions allow positioning near neurites and somas with minimal perturbation, and integration with guided neuron‐growth patterns enabled correlation of structural connectivity with electrical activity, illustrating how nanoscale transistors can interrogate organized neuronal circuits. Beyond extracellular sensing, nanowires can also directly enter cells. Fu et al. engineered sub‐10 nm nanowire‐nanotube heterostructures capable of intracellular access with extremely limited invasiveness [153]. These heterostructures recorded intracellular electrical signals in cardiomyocytes while maintaining cell viability, demonstrating the feasibility of distributed intracellular electrophysiology with nanoscale electrodes. Although this work focused on cardiomyocytes, similar design principles could be extended to neurons, offering a route toward minimally invasive, high‐resolution intracellular neural recording [154]. Such approaches alleviate some geometric and mechanical limitations of patch‐clamp electrodes, enabling simultaneous recording from multiple cells.

Together, these studies underscore the importance of nanoscale curvature, membrane mechanics, and surface chemistry in enabling stable cellular access, positioning nanowire‐based technologies as a key component of nanoscale brain monitoring by achieving spatial resolutions and membrane coupling not attainable with larger electrodes. Collectively, mesh electronics, nanowire‐based probes, and ultraflexible arrays highlight the evolution of nanoelectronics toward chronic, high‐density neural recordings [151]. Unlike traditional microelectrodes, these nano‐enabled interfaces are explicitly designed to merge with brain tissue rather than simply penetrate it. Their reduced chronic immune response, geometric adaptability, and subcellular coupling make them central tools for precision electrophysiological mapping, capable of tracking long‐term brain states and neural circuit dynamics with unprecedented resolution.

### 7.2 Optical and Electrochemical Nanosensors for Chemical and Ionic Monitoring

Neural computation is shaped not only by fast electrical signaling, but also by dynamic fluctuations in neuromodulators and ions that regulate circuit excitability, plasticity, and behavioral state. Monitoring these biochemical variables in vivo has historically been restricted by poor selectivity, photobleaching, tissue scattering, and invasiveness of conventional electrochemical approaches. Recent developments in nanoscale chemical sensors, particularly optical nanosensors based on functionalized SWCNTs, nanostructured electrochemical electrodes, and upconversion‐enabled ion probes, address these limitations by exploiting the high surface‐area‐to‐volume ratios, tunable surface chemistry, and mechanical compliance that emerge at the nanoscale. These properties enable minimally invasive probes capable of stable, real‐time chemical imaging deep within intact brain tissue.

**FIGURE 7:**
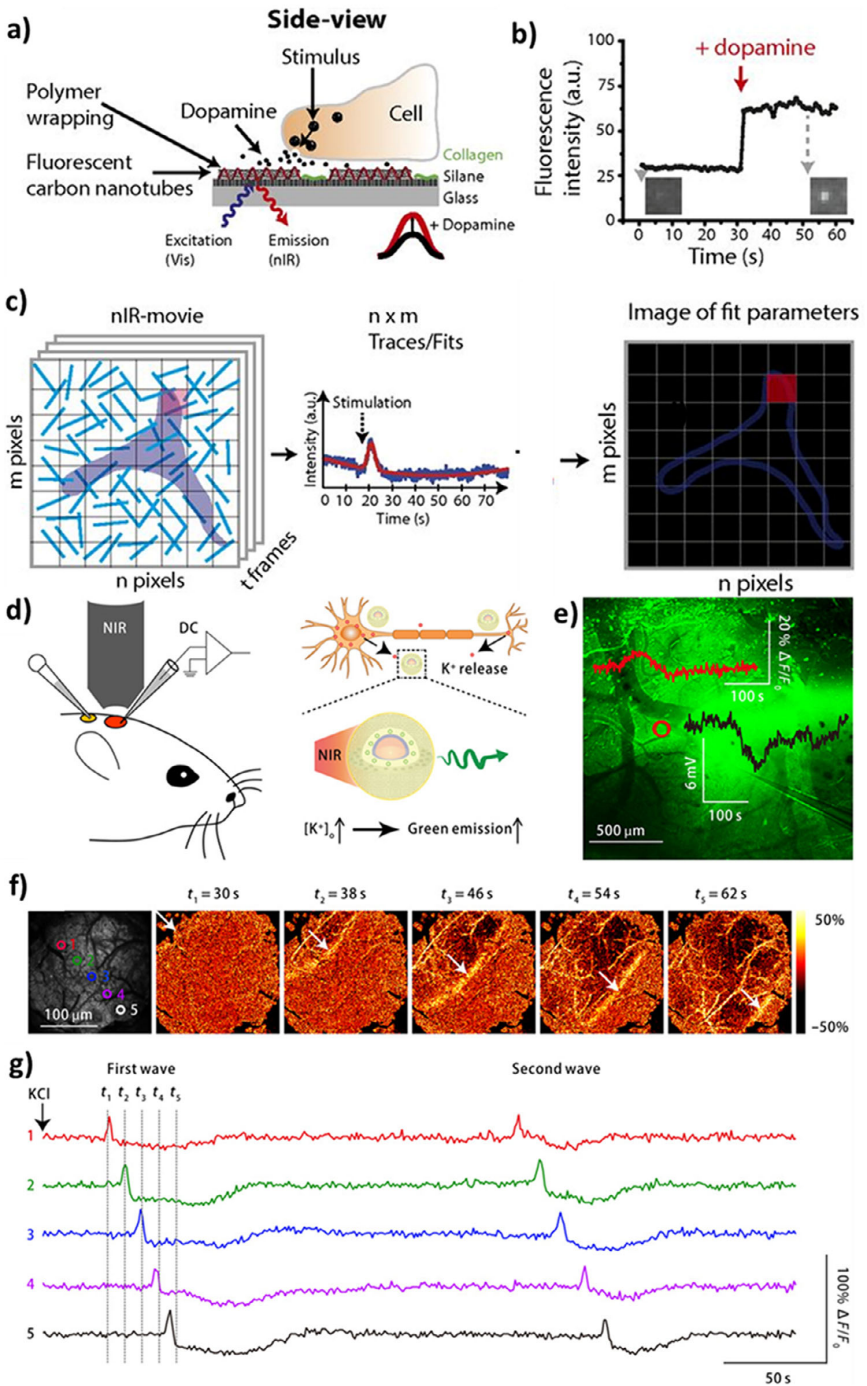
Fluorescent nanosensor imaging of neurotransmitter and ion dynamics. (a–c). Chemical imaging using high‐density fluorescent nanosensor arrays. (a) Dopamine‐sensitive fluorescent SWCNTs, wrapped with specific ssDNA sequences, immobilized on glass substrates, and interfaced with dopamine‐releasing PC12 cells. Dopamine binding modulates SWCNT NIR emission. (b) Representative fluorescence trace from a single nanosensor responding to 10 µm dopamine. (c) Pixel‐wise analysis of NIR movies enables extraction of quantitative kinetic parameters and generation of spatial maps of dopamine release. Adapted from [157], 2017, National Academy of Sciences. (d–g). Nanosensor imaging of extracellular K^+^ dynamics in a cortical spreading depression (CSD) murine model. (d) Schematic of combined optical and electrophysiological monitoring of CSD in the thinned‐skull mouse cortex. (e) Surface fluorescence image showing nanosensor responses to CSD‐evoked K^+^ elevation, with corresponding local field potential trace. (f) Time‐lapse pseudo‐color images depicting propagation of the K^+^ wave. (g) Fluorescence time courses from selected cortical regions, illustrating sequential activation during wave propagation. Adapted from [159], 2019, AAAS.

Representative examples of nanosensor imaging of neurotransmitter and ion dynamics are shown in Figure 7. Among optical tools, SWCNTs have emerged as powerful NIR probes due to their photostable emission and sensitivity to molecular binding when functionalized on their surface with polymers or DNA sequences. Early work showed that SWCNT fluorescence can be modulated by specific chemical interactions, creating synthetic recognition sites on their surfaces [155]. Building on this principle, Beyene et al. engineered a nongenetically encoded NIR dopamine nanosensor by wrapping SWCNTs with synthetic polymers whose corona‐phase conformations change upon catecholamine binding, producing robust fluorescence modulation suitable for deep‐brain imaging. In vivo experiments in the mouse striatum showed that SWCNT nanosensors could detect electrically and optogenetically evoked dopamine release with high temporal fidelity and minimal scattering, demonstrating their compatibility with complex neural tissue and their ability to report sub‐second neuromodulator fluctuations [156]. Complementary work by Kruss et al. expanded the spatial resolution of SWCNT‐based chemical sensing through the development of high‐density fluorescent nanosensor arrays capable of sampling dopamine efflux at unprecedented spatial resolution. Using DNA‐wrapped SWCNTs engineered through corona phase molecular recognition, they achieved highly selective dopamine detection without genetic modification, enabling the construction of arrays with more than 20 000 sensors per cell. These arrays revealed steep concentration gradients, heterogeneous release sites, and spatial microdomains of neuromodulator signaling that were previously inaccessible with microdialysis or fast‐scan cyclic voltammetry (Figure 7a–c) [157].

Together, these optical nanosensors demonstrate how nanomaterial functionalization allows molecular specificity, deep‐tissue NIR detection, and minimal invasiveness, enabling chemical imaging at spatial and temporal scales matched to neural computation. Electrochemical nanosensors complement these optical systems by offering exceptional temporal resolution, rapid electron‐transfer kinetics, and compatibility with established neuromodulatory measurement techniques such as fast‐scan cyclic voltammetry. Carbon nanotube yarn microelectrodes exemplify this approach. Mendoza et al. demonstrated that twisting carbon nanotubes into nanoscale yarns dramatically increases surface roughness, electroactive surface area, and conductivity, enabling rapid electron transfer and enhanced sensitivity to dopamine and serotonin [158]. These electrodes exhibited low fouling, stable long‐term performance, and high signal‐to‐noise ratios in vivo, enabling reliable detection of sub‐second monoamine transients at micrometer‐scale sensor dimensions. Their mechanical durability and resistance to biofouling make carbon nanotube yarns promising candidates for chronic neurochemical interfacing.

Beyond neurotransmitters, nanoscale probes enable direct imaging of ionic fluctuations that modulate excitability, seizure propagation, and spreading depolarizations. Conventional ion indicators suffer from limited selectivity, shallow imaging depth, and phototoxicity. Liu et al. addressed these challenges by engineering a UCNP‐based K^+^ nanosensor that integrates NIR excitation, a K^+^‐selective membrane, and luminescence resonance energy transfer between UCNPs and the K^+^ indicator potassium‐binding benzofuran isophtalate (Figure 7d–g) [159]. This design minimizes autofluorescence, permits deep‐tissue imaging, and produces highly sensitive, reversible K^+^ readouts. Importantly, the K^+^‐selective membrane not only excludes competing cations but also concentrates K^+^ within the nanosensor, amplifying physiologically relevant fluctuations. These capabilities enabled detection of extracellular K^+^ dynamics in the intact mouse brain, providing access to ionic signals underlying pathological events such as spreading depolarizations.

Together, these nanosensors represent a significant expansion of the chemical monitoring landscape. Optical SWCNT probes achieve deep‐tissue neuromodulator imaging with unmatched photostability, nanostructured electrochemical electrodes detect monoamines with millisecond precision, and upconversion‐based ion nanosensors reveal dynamic ionic environments previously inaccessible with classical indicators. Beyond device performance, these nanoscale tools are reshaping biological understanding by exposing microdomain‐specific neurotransmitter release, heterogeneous uptake, and propagating ionic waves during pathological events. As with nanoelectronic probes, their nanoscale sizes promote minimal perturbation of brain tissue, and continued improvements in antifouling coatings, biocompatible functionalization, and chronic stability suggest the possibility of long‐term chemical monitoring. In the context of precision brain interfacing, these optical and electrochemical nanosensors provide a multidimensional view of brain chemistry, enabling concurrent monitoring of electrical, neuromodulatory, and ionic signals that collectively govern neural computation.

Despite rapid progress, several challenges must be addressed for nanosensors to reach their full potential as long‐term, multimodal interfaces with the brain. A major obstacle is the delivery and spatial targeting of nanoscale probes. Ultrathin nanoelectronic devices can be implanted with micrometer precision; however, optical and electrochemical nanosensors, particularly freely diffusing constructs, are more challenging to localize to defined cell types, and mislocalization increases the risk of inflammation or disruption of tissue architecture [1, 160]. Achieving stable placement over months will require new strategies for anchoring, scaffold‐based integration, or activity‐dependent immobilization [161]. Long‐term stability also remains a central concern. Although nanoelectronic meshes and threads evoke minimal glial scarring, the chronic performance of chemical nanosensors can degrade due to biofouling, loss of functional coatings, or intracellular and extracellular accumulation of nanomaterials [162]. Advances in antifouling surface chemistries, durable and regenerable ligand or polymer functionalization, and controlled biodegradation or clearance pathways will be essential for maintaining reliable sensing over extended timescales. A further frontier lies in multimodal and multiplexed integration. Simultaneously measuring electrical activity, neurotransmitter release, and ionic dynamics in a unified nanoscale platform would offer an unprecedented view of how neurons integrate these modalities to perform neural computation, yet achieving this goal without compromising device size, flexibility, or biocompatibility remains technically demanding. Solutions will likely require co‐design of materials, architectures, and readout modalities at the nanoscale. Finally, clinical translation will depend on a comprehensive evaluation of long‐term biocompatibility, immune responses, material clearance pathways, and manufacturing scalability. Nonetheless, the minimal invasiveness, high sensitivity, and tunable physicochemical properties of nanosensors position them as promising components of next‐generation precision neurotechnologies, tools capable of capturing the intertwined electrical and chemical signals that underlie brain function and dysfunction. Beyond device‐level performance, the translational viability of nanoscale brain interfaces will also depend on whether probes, transducers, and payloads can be delivered across or around the brain's protective barriers with sufficient spatial precision and quantitative control.

## 8 Crossing the Barriers: Strategies for Efficient Delivery and Targeting

**FIGURE 8:**
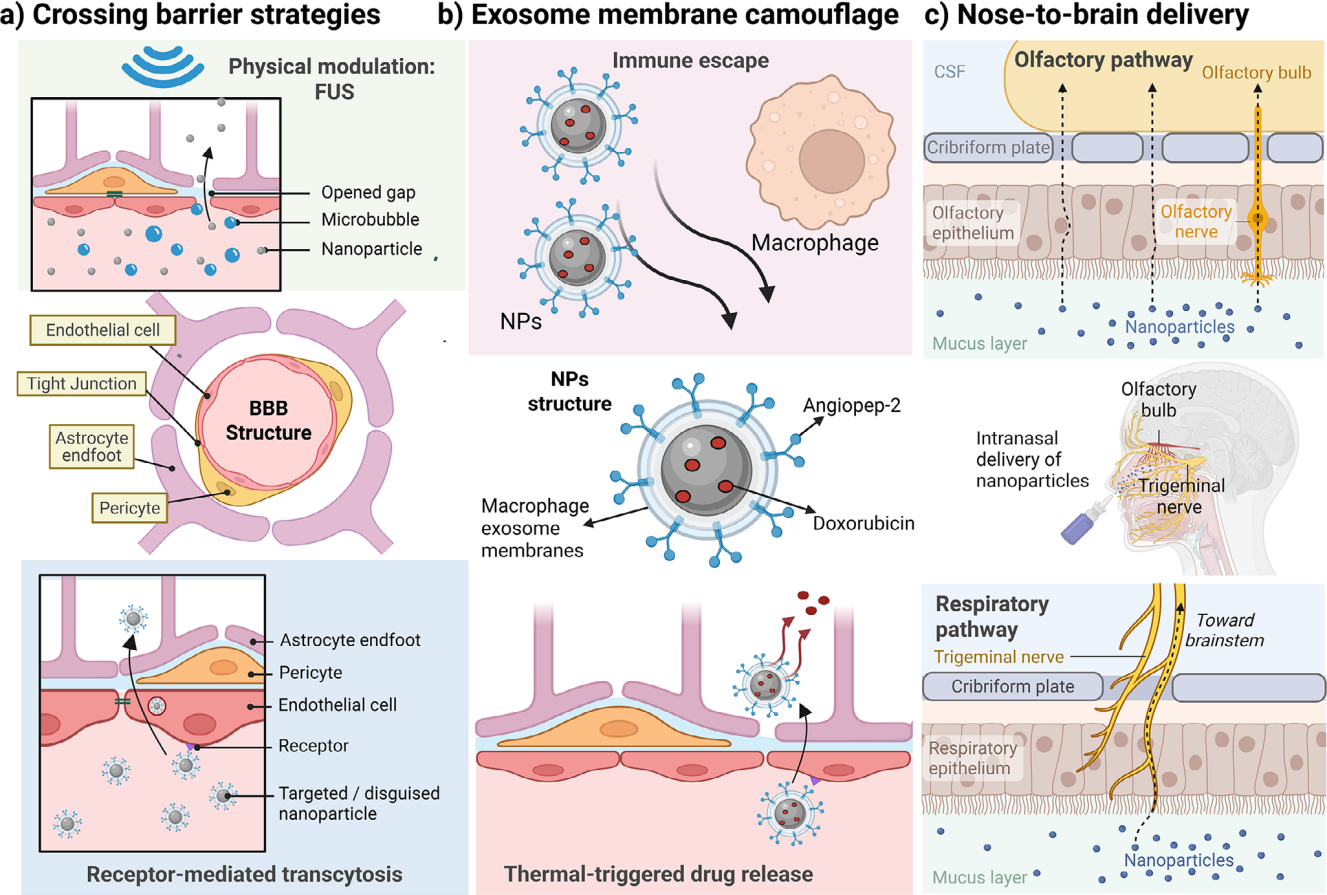
Schematic overview of nanotechnology‐enabled strategies for crossing brain barriers. (a) BBB structure and physical or biological modulation strategies, including FUS with microbubbles to transiently open tight junctions and RMT of targeted or disguised nanoparticles. (b) Exosome‐membrane camouflage, where nanoparticles cloaked with macrophage‐derived membranes evade immune clearance and can be further functionalized (e.g., with angiopep‐2) and loaded with a drug (e.g., doxorubicin) for BBB targeting and thermal‐triggered drug release. (c) Nose‐to‐brain delivery routes in which intranasally administered nanoparticles traverse the olfactory and respiratory epithelia to reach the CNS via the olfactory bulb and trigeminal nerve pathways. Created with BioRender.com.

Crossing the protective interfaces of the CNS remains one of the central challenges in precision neurotherapeutics and smart brain‐interfacing technologies (Figure 8). The impermeability of the BBB, reinforced by stringent neurovascular and glial interfaces, preserves homeostasis while severely restricting delivery of therapeutic and diagnostic agents. This complex cell network, formed by endothelial cells with tight junctions, astrocytic endfeet, pericytes, and a finely regulated basement membrane, constitutes a dynamic but highly selective filter that evolutionarily prioritizes neural protection over molecular accessibility [163]. Although the last decade has witnessed an accelerated expansion of strategies aimed at enhancing brain delivery, true precision, defined as predictable, controllable, and cell‐type‐specific transport, remains elusive. Many recent advances demonstrate technical feasibility yet still fall short of the mechanistic clarity and translational predictability required for clinical neurotechnology. As a result, understanding both the strengths and the persistent gaps within current strategies is essential to charting a realistic path forward and designing next‐generation systems capable of interfacing with the brain in ways that are both effective and safe [18].

Physical modulation of barrier permeability continues to be among the most mature strategies. The recent multifunctional theranostic ultrasound platform exemplifies the sophistication reached by FUS, coupling microbubble‐mediated mechanical opening with real‐time image guidance and enabling brain‐wide delivery of SPIONs and viral vectors encoding thermoreceptors for remote magnetogenetics [164]. This study demonstrates that the pairing of acoustic energy with circulating nanocarriers can expand the spatial distribution of delivered agents while maintaining a degree of regional selectivity that would be unattainable through systemic administration alone.

Despite these remarkable demonstrations, the assumption that transient BBB opening is biologically neutral remains insufficiently examined. Subtle inflammatory responses, endothelial stress, microhemorrhages, and long‐term vascular remodeling remain under‐characterized, making it difficult to assess the cumulative impact of repeated FUS sessions. Moreover, translation to human anatomy, where skull thickness, vascular heterogeneity, and microbubble behavior markedly differ from rodents, raises uncertainties that current studies rarely address. Even within the same species, disease‐dependent BBB alterations complicate the reproducibility of FUS‐mediated delivery, particularly in regions compromised by tumors, neurodegeneration, or inflammation [164]. The technology is compelling, but not immune to questions regarding long‐term safety and consistency across patients.

Conversely, strategies exploiting endogenous transport mechanisms appear more physiological but are accompanied by their own limitations. RMT remains the conceptual backbone of BBB‐targeting nanomedicine. The ApoE‐corona oncolytic adenovirus exemplifies how nanocarriers can exploit endogenous proteins and receptors to achieve both BBB penetration and glioblastoma targeting. In this system, ApoE recruitment enables LRP1 engagement and promotes immunogenic tumor cell death [165]. This approach highlights a crucial shift: rather than forcing passage across the barrier, it co‐opts natural ligand‐receptor pathways to shuttle therapeutic entities into the brain. Similarly, dual‐responsive choline transporter‐targeting nanoparticles combine RMT with intracellularly triggered drug activation, offering a rational two‐stage traversal of the endothelial and tumor microenvironments [166]. These platforms advance the field significantly, yet RMT is inherently saturable and highly sensitive to receptor expression levels, ligand affinity, and endosomal trafficking dynamics. The mechanistic underpinnings of nanoparticle fate during transcytosis remain surprisingly opaque, and most studies infer BBB crossing from downstream accumulation rather than direct visualization of the cellular transport process [166]. Furthermore, human BBB receptor profiles substantially differ from those of rodent models, raising legitimate concerns about the predictive validity of preclinical data and the scalability of these strategies for therapeutic use.

LNPs and nucleic acid delivery platforms have recently gained momentum, reflecting the broader success of LNPs in systemic gene therapy. Their application to CNS delivery has been bolstered by FUS‐mediated assistance, which has been shown to enhance the delivery of SOD1 antisense oligonucleotides for amyotrophic lateral sclerosis models and to enable efficient RNA transport to glioblastoma using low‐frequency [167, 168]. High‐throughput in vivo screening platforms have begun to reveal structure‐function rules governing brain‐targeted LNP formulations [169], and subtle modifications such as PEG‐lipid anchor length exhibit meaningful influence on siRNA brain uptake [170]. Despite these gains, LNP biodistribution remains dominated by hepatic and splenic sequestration, and CNS‐directed optimization is still largely empirical. Predictive models for LNP‐BBB interaction are lacking, and scaling from small to large species remains a significant challenge that requires deeper mechanistic insight.

Across all delivery modalities, the need for quantitative, mechanistically informative readouts is becoming increasingly evident. Imaging strategies such as positron emission tomography monitoring of 89Zr‐Talidox following FUS‐mediated BBB opening offer glimpses into more rigorous pharmacokinetic tracking [171]. Many studies still rely on relative fluorescence or *ex vivo* biodistribution without establishing absolute delivered doses, time‐dependent transport profiles, or cell‐type resolution. Without standardized metrics, cross‐platform comparisons remain speculative, and claims of enhanced delivery are often difficult to contextualize.

Bioinspired systems, particularly those relying on cell membrane camouflaging, seek to endow synthetic carriers with biological intelligence. Exosome‐membrane‐disguised thermoresponsive nanoplatforms are a prime example, combining the natural tropism and immune compatibility of extracellular vesicles with engineered thermal‐triggered release across the BBB [172]. These hybrid constructs take advantage of the unique protein and lipid signatures of exosomal membranes, enabling them to evade immune clearance and engage receptor‐mediated interactions that improve their brain‐homing potential. While conceptually elegant, such systems face challenges in reproducibility, batch‐to‐batch consistency, and precise molecular characterization of the membrane components responsible for targeting [172]. The biological complexity that makes these carriers appealing also introduces uncertainty, and the heterogeneity of exosomal membranes complicates regulatory translation. Nevertheless, their ability to integrate stealth, targeting, and responsiveness makes them one of the most promising directions for next‐generation neurotherapeutics.

Alternative anatomical routes, such as nose‐to‐brain delivery, provide a non‐invasive bypass to the BBB and have shown renewed promise. Biomineralized silk‐fibroin nanoparticles demonstrated potent neuroinflammation modulation and alleviation of depressive‐like symptoms via olfactory and trigeminal transport pathways [173]. However, the inherent variability of nasal anatomy and mucociliary clearance limits reproducibility, and dose scaling to larger species remains unresolved. While promising as an adjunct or for specific indications, nose‐to‐brain strategies may face difficulties achieving the spatial precision and quantitative control required for the broader spectrum of neurotechnological applications.

Altogether, while the field has achieved remarkable progress, ranging from ultrasound‐expanded permeability and receptor‐guided transport to exosome‐disguised carriers and LNP‐based genetic delivery, precision brain interfacing continues to advance through fragmented, partially overlapping strategies rather than a unified framework. A deeper mechanistic understanding of barrier dynamics, combined with standardized quantitative methodologies and careful consideration of translational constraints, will be essential to transform these diverse innovations into reliably predictable, clinically meaningful brain‐targeting technologies.

## 9 Future Directions and Clinical Roadmap

Taken together, these delivery and targeting considerations underscore that efficient access to the brain is a central translational requirement for smart nanotechnologies, and should therefore be viewed as a key determinant of their future clinical implementation. The examples surveyed in this review collectively suggest that precision brain interfacing is no longer a purely conceptual goal but an emerging technological domain with identifiable translational trajectories. Soft mesh nanoelectronics that conform to brain organoids and neural tissue, organic and inorganic nanotransducers capable of wireless stimulation, and nanosensors that report electrophysiological and chemical activity already demonstrate stable operation in complex biological environments over weeks to months. In the near term, the clearest path to the clinic lies in indications where local access is surgically routine, risk‐benefit ratios are favorable, and readouts are unambiguous. Vision restoration is a prominent example. Subretinally injected semiconducting polymer nanoparticles that act as a “liquid prosthesis” have restored visually guided behaviors in rodent models of retinitis pigmentosa, with efficacy approaching that of current electronic prostheses but without rigid implants or external cameras [112]. Similarly, broadband TeNWNs and plasmonic nanorods point to retinal interfaces that extend sensitivity into the NIR while remaining wire‐free [117]. Together, these systems define a plausible translational route: minimally invasive injections into a confined compartment, optical activation via external hardware already familiar in ophthalmology, and visual endpoints that can be quantified behaviorally and psychophysically.

Regarding precision deep‐brain stimulation, advances in nanotransducers for wireless neuromodulation, like magnetothermal, photothermal, photoelectrochemical, and piezoelectric mechanisms, are beginning to chart design rules for safe power densities, effective volumes of influence, and multimodal actuation [174]. Piezoelectric nanogenerators that rescue dopaminergic signaling and motor behavior in Parkinsonian models under ultrasound activation show how such platforms can be tuned for disease‐specific neuromodulation while remaining mechanically and chemically compatible with neural tissue [175].

At the same time, the field is converging with the broader effort to develop flexible, tissue‐compliant brain interfaces. Ultraflexible electrode arrays that provide months‐long, high‐density recording from thousands of neurons in rodents, and stretchable, mesh‐like architectures that conform to brain curvature and organoid expansion, demonstrate that mechanical matching and nanoscale cross‐sections can markedly extend functional lifetimes of implanted electronics [176]. Conceptual frameworks for flexible BMIs now explicitly call for integration of soft electronics with advanced materials, wireless power and data links, essential ingredients for clinically relevant, chronic BMIs [177]. Smart nanomaterials will increasingly be embedded into such architectures as local transducers and chemical sensors rather than free colloids, simplifying regulatory classification and enabling precise mapping among device geometry, dose, and effect.

Despite rapid progress, several cross‐cutting challenges must be solved before smart nanotechnologies can be deployed as routine clinical tools. First, safety and biostability must be demonstrated on human‐relevant timescales. For many nanotransducers, the relevant horizon is not weeks but years. Chronic data on accumulation, degradation products, and long‐term immune consequences remain scarce, particularly for inorganic materials such as magnetoelectrics and chalcogenide semiconductors. Recent reviews emphasize that even flexible macro‐scale BMIs require careful engineering of material interfaces to mitigate glial scarring, micromotion‐induced damage, and corrosion; these considerations will be at least as stringent for nano‐enabled systems that are more difficult to retrieve once deployed [178]. A pragmatic near‐term strategy is to prioritize transient or partially degradable platforms like organic semiconductors, bioresorbable conductors, and degradable piezoelectrics, that can deliver a defined period of function followed by controlled elimination, analogous to resorbable sutures. Second, energy delivery and dose control must be reframed in clinical language. For optical and magnetic modalities, preclinical work is beginning to define safe exposure limits and therapeutic windows, but translation will require harmonizing these with existing guidelines for photomedicine, magnetic resonance imaging, and neuromodulation. Comprehensive reviews of optical neuromodulation have underscored the importance of accounting for wavelength‐dependent scattering, heating, and photochemical effects, especially when operating in the NIR windows favored for deep tissue penetration [179]. For FUS, which underpins many sono‐genetic and sono‐responsive platforms, clinical experience in thermoablation and BBB opening already provides a rich safety and dosimetry framework. A similar codification is now needed for magnetoelectric and piezoelectric neuromodulation: standardized reporting of field strengths, duty cycles, local temperature changes, and induced potentials will enable analyses and comparison across platforms. Third, scalable manufacturing and regulatory alignment will determine whether smart nanotechnologies remain laboratory tools or mature into approved therapies. Many of the examples highlighted in this review rely on multi‐step syntheses, delicate self‐assembly, or biologically derived coatings that are challenging to standardize. Bridging the gap to good manufacturing practice will require early engagement with chemistry, manufacturing, and control experts, as well as simplification of architectures wherever possible. Combination‐product pathways, where nanotransducers are integrated into flexible electrodes, hydrogels, or catheters, may prove more tractable than stand‐alone injectable nanoparticles, because they more naturally align with existing device regulations and quality systems developed for bioelectronic medicine.

The route from preclinical demonstration to first‐in‐human trial will also depend on access to predictive experimental platforms. Here, organoid‐on‐chip systems with embedded nanoelectronics provide a powerful bridge between animal models and human physiology, enabling long‐term assessment of efficacy, toxicity, and network‐level impact in human‐derived tissue [180]. Such platforms can be used to iteratively optimize nanomaterial composition, geometry, and stimulation paradigms before committing to invasive studies in large animals.

In the near term, the most promising clinical applications are those with confined and accessible targets, such as the retina, or highly debilitating pathological conditions with rapid progression and poor prognosis, such as brain tumors and certain neurodegenerative disorders. Nanomaterial design must be aligned with safe energy delivery, biointegration, scalable manufacturing, and regulatory requirements. If these components can be coordinated, precision brain interfacing may quickly progress to clinics for diagnosing and treating disorders of the nervous system.

## Author Contributions

**Tommaso Curiale**: conceptualization, visualization, writing – original draft, writing – review and editing. **Marie Celine Lefevre**: conceptualization, visualization, writing – original draft. **Alessio Carmignani**: visualization, writing – original draft. **Maria Cristina Ceccarelli**: visualization, writing – original draft. **Matteo Battaglini**: visualization, writing – original draft. **Attilio Marino**: conceptualization, visualization, supervision, writing – original draft, writing – review and editing. **Gianni Ciofani**: conceptualization, supervision, project administration, resources, writing – review and editing.

## Conflicts of Interest

The authors declare no conflicts of interest.

## Data Availability

No data have been produced for this work.
